# Epigenetic regulation in oogenesis and fetal development: insights into m6A modifications

**DOI:** 10.3389/fimmu.2025.1516473

**Published:** 2025-04-28

**Authors:** Lusheng Liu, Danxia Ge, Yumeng Lin, Zhongyu Han, Heng Zhao, Liqin Cao, Xi Wu, Guizhi Ma

**Affiliations:** ^1^ Department of Acupuncture and Moxibustion, Shanghai Traditional Chinese Medicine (TCM)-Integrated Hospital, Shanghai University of Traditional Chinese Medicine, Shanghai, China; ^2^ Shanghai Clinical Medical College of Integrated Traditional Chinese and Western Medicine, Shanghai University of Traditional Chinese Medicine, Shanghai, China; ^3^ Department of Critical Care Medicine, Traditional Chinese Medicine Hospital of, Ningbo, Zhejiang, China; ^4^ Nanjing Tongren Hospital, School of Medicine, Southeast University, Nanjing, China; ^5^ Shanghai TCM-Integrated Hospital, Shanghai University of Traditional Chinese Medicine, Shanghai, China; ^6^ Department of Gynecology, Shanghai TCM-Integrated Hospital, Shanghai University of Traditional Chinese Medicine, Shanghai, China

**Keywords:** RNA modification, epigenetic modification, female reproductive diseases, reproductive system neoplasms, treatment

## Abstract

The unique physiological structure of women has led to a variety of diseases that have attracted the attention of many people in recent years. Disturbances in the reproductive system microenvironment lead to the progression of various female tumours and pregnancy disorders. Numerous studies have shown that epigenetic modifications crucially influence both oogenesis and foetal development. m6A, a modification at the mRNA level, consists of three parts, namely, writers, erasers, and readers, which are involved in several biological functions, such as the nucleation and stabilisation of mRNAs, thereby regulating the development of reproductive system diseases. In this manuscript, we delineate the constituents of m6A, their biological roles, and advancements in understanding m6A within the maternal–foetal immunological context. In addition, we summarise the mechanism of m6A in gynaecological diseases and provide a new perspective for targeting m6A to delay the progression of reproductive system diseases in clinical practice.

## 1 Introduction

In recent years, the incidence of female reproductive system diseases has steadily increased, and these diseases have become a common problem worldwide, seriously affecting women’s quality of life (1). Common gynaecological diseases include benign diseases, such as endometriosis, premature ovarian failure, and adenomyosis; diseases during pregnancy, such as preeclampsia, recurrent miscarriage, and gestational diabetes; and malignant tumours, such as cervical cancer (CC), ovarian cancer (OC), and endometrial cancer (EC). Therefore, there is an urgent need to understand the pathogenesis of female reproductive system diseases and find specific biomarkers, so as to improve the quality of life of women.

The N6-methyladenosine (m6A) modification, a form of internal chemical alteration within RNA, was first identified in the 1970s. This process involves the methylation of the nitrogen atom at the 6th position of the adenine (A) base within RNA, catalysed by methyltransferases. This modification is prevalent in both messenger RNA (mRNA) and non-coding RNA (ncRNAs) (2). Through writers, erasers, and readers, m6A is implicated in diverse mechanisms and metabolic processes, including pre-mRNA splicing, export, and translation; the regulation of translation efficiency; mRNA stability; the prevention or delay of mRNA degradation; and the processing of noncoding RNAs (3–5). M6A is associated with female reproductive system function, and some abnormal m6A modifications have also been observed to be involved in the generation, metastasis, and drug resistance of cancer cells (6). Further studies revealed that m6A methylation affects mainly carcinogenesis and malignant tumour progression by regulating gene expression in cancer (7, 8). Thus, the potential association of aberrant m6A with cancer was documented.

This article reviews the latest research progress on the biological role of m6A in gynaecological tumours and pregnancy-related diseases and discusses the mechanism of m6A regulatory proteins in the proliferation, invasion and metastasis of gynaecological tumours. This article also discusses the research progress and future directions of m6A modification in the maternal–foetal immune environment and the diagnosis and prognostic evaluation of gynaecological diseases, with the aim of revealing the potential role of RNA methylation in female reproductive system diseases to gain a deeper understanding of these diseases.

## 2 Mechanisms and regulation of m6A RNA methylation

### 2.1 Writers

**Figure 1:**
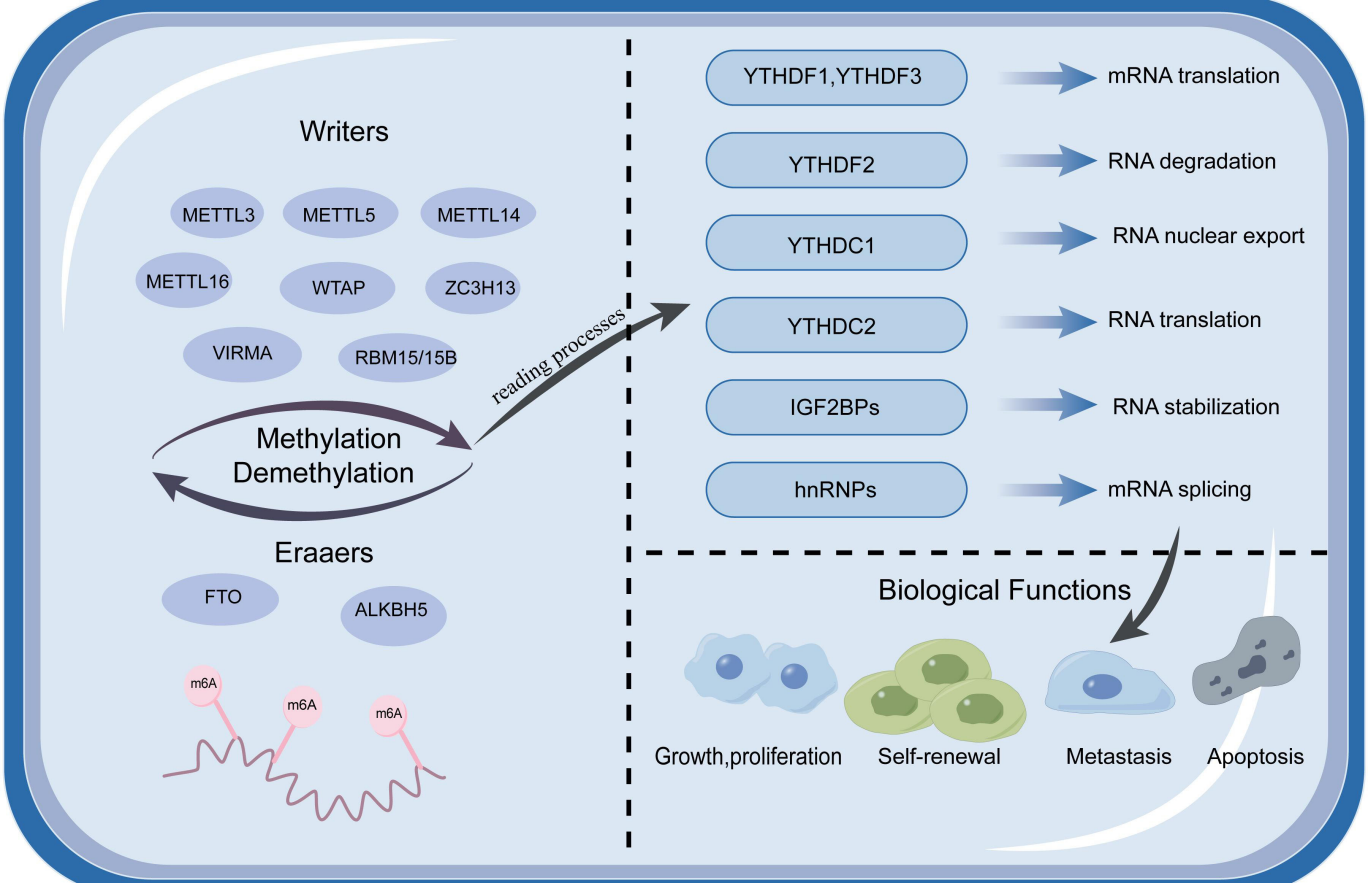
Shows the regulatory mechanism of m6A modification, which consists of three major parts and elaborates its working principle. N6-methyladenosine (m6A) is methylated by a methyltransferase complex containing METTL3, METTL14, WTAP, and KIAA1429, and these proteins called “writers” are responsible for adding the m6A modification; while “erasers” FTO and ALKBH5 are responsible for reversibly removing m6A. The reader proteins YTHDC1/2, YTHDF1/2/3, and IGF2BP1/2/3 are responsible for performing the biological functions of m6A, including translation, decay, splicing, and post-transcriptional regulation of RNA.

The m6A writer is a methyltransferase with dynamic, reversible properties during the formation process and many proteins function as writers, including methyltransferase-like3(METTL3), methyltransferase-like5 (METTL5), methyltransferase-like 14 (METTL14), methyltransferase-like 16 (METTL16), Wilms tumour type 1 associated protein (WTAP), Vir-like m6A methyltransferase-associated protein (VIRMA), RNA-binding motif 15/15B (RBM15/15B, zinc finger CCCH-containing type 13 (ZC3H13, KIAA0853), and methyltransferase-like 16 (METTL16) (9–11) (**Figure 1**).

METTL3 was the first methyltransferase shown to be involved in m6A methylation in 1997, and its subunits play critical roles in the catalytic process to facilitate the transfer of adenine from S-adenosylmethionine (SAM) moieties to RNA (12). Until 2017, there were relevant studies on the role of METTL3 as an m6A methyltransferase in human cancer. It has been shown that METTL3 acts as an oncogene in AML and its activity depends on interaction with the CAAT-box binding protein CEBPZ, which promotes METTL3 localisation to chromatin and induces m6A modification on specific mRNA transcripts such as SP1 and SP2 (13).

The oncogene role of METTL3 in AML has been well-established and its overexpression and oncogenic activity have subsequently been revealed in a variety of cancer types, including hepatocellular carcinoma, gastric cancer, and non-small cell lung cancer (14–17). In gastric cancer, elevated expression levels of METTL3 were demonstrated to be an independent poor prognostic indicator (18). In bladder cancer, METTL3 overexpression has been shown to be associated with unfavourable prognostic outcomes, and this overexpression promotes methylation of specific mRNA molecules (19). In the female reproductive system, overexpression of METTL3 is thought to have a role in promoting oocyte maturation. In the genital vesicle stage of oocytes, the loss of function of Mettl3 severely inhibits meiotic maturation of oocytes, and its inactivation may lead to abnormal oocyte development, affect the stability of ovulation and meiosis, and then cause reproductive system diseases such as infertility (20).

METTL14 and METTL3 are molecular homologues that form stable heterodimeric complexes, and METTL14 can promote RNA recognition and binding by stabilising the conformation of METTL3, providing structural support, and enhancing its catalytic activity (21–25). METTL14 was initially found to be overexpressed in acute myeloid leukaemia and plays a key oncogenic role in the initiation and development of the disease (26). METTL14 has also been shown to be downregulated and exhibit tumour suppressive effects in some cancers. In patients with endometrial cancer, METTL14 levels are decreased in tumour samples, promoting the proliferation of endometrial cancer cells (27).

It has been shown that in hepatocellular carcinoma, down-regulation of METTL14 and m6A expression is significantly associated with the high metastatic ability of tumours and the prognosis of patients (28). In breast cancer, the expression levels of METTL14 and ALKBH5 regulate each other and together promote tumour development by regulating m6A modification levels in specific transcripts associated with epithelial-mesenchymal transition (EMT) and angiogenesis (29).

WTAP is a ubiquitously expressed nuclear protein whose transcription is regulated by WT-1 and interacts with the METTL3/METTL14 complex to affect m6A RNA methylation, thereby regulating gene transcription and RNA splicing. WTAP regulates transcription and translation of niche factors by placing m6A markers directly or indirectly on transcripts encoding niche factors (30). In acute myeloid leukaemia, WTAP has been shown to be up-regulated at its expression level and plays an important role in the proliferation and differentiation of tumours (31). In a zebrafish embryo model, a decrease in WTAP or METTL3 expression levels results in abnormal tissue differentiation and increased rates of apoptosis (9). WTAP has been identified as a prototypical biomarker of OC development and spread (32, 33). Liu et al. found that WTAP expression was higher in EC tissues than in surrounding normal tissues (34).

As an RNA-binding protein, RBM15 and RBM15B promote the recognition and assembly of mRNA molecules containing specific sequences by METTL3 and WTAP through their RNA-binding domains, which is critical for the precise regulation of m6A methylation. HAKAI protein plays a bridge role in this process, which mediates the binding of mRNA to methylation complexes and participates in the regulation of mRNA degradation. In particular, HAKAI ensures the stability of the sequence characteristics and functional status of mRNAs after m6A methylation, which has a crucial role for the accurate transmission of genetic information and the function of mRNAs in subsequent cellular processes, such as protein translation.

KIAA1429 can bind to WTAP, mediate the formation of the METTL3/METTL14/WTAP complex, preferentially bind to the 3’UTR and participate in alternative polyadenylation (APA) while preventing target RNA coding through selective methylation (10, 35).

METTL16 is a catalytically active m6A methyltransferase that regulates the homeostasis of the SAM content via the splicing of the U6 snRNP (36). METTL16 binds directly to MAT2A mRNA precursors, which have a conserved hairpin structure in the 3 ‘untranslated region (UTR) and affect alternative splicing of MAT2A and homeostasis of SAM content in cells. In addition, in mice, METTL16 also regulates SAM synthase expression, which in turn impacts early embryonic development (37, 38). Previous studies have shown that METTL16 expression levels are upregulated in multiple cancer types and are associated with poor prognosis, including gastric cancer, hepatocellular carcinoma, pancreatic cancer, and acute myeloid leukaemia (39–43).

### 2.2 Erasers

The m6A eraser is a demethylase that removes m6A methylation from the targeted RNAs, unlike other m6A proteins, and only the AlkB homologue 5 (ALKBH5) protein and fat mass and obesity-associated (FTO) protein have been identified as erasers (44–46).

FTO, as the first m6A demethylase identified, was examined and assigned to the AlkB family. It has the ability to remove m6A as well as n6, 2^′^ -O-dimethyladenosine (5^′^cap m6A_m_) from the mRNA interior, thereby affecting m6A modification (47, 48). In addition, FTO localises predominantly in the nucleus and regulates about 10% of m6A modifications in all cell lines (49, 50). Recently, the discovery of the demethylase FTO revealed its reversible control of the kinetics of the m6A methylation process under physiological and pathological conditions (47, 48).

In CC, the interaction of FTO with E2F1 and MYC significantly decreased their translation efficiency. When E2F1 or MYC expression levels are elevated, they are able to complement the loss-of-function of FTO and have negative effects on cell proliferation and migration, suggesting that these two genes may play a mutually regulated role in CC cells (51). Wang and colleagues have suggested that FTO influences the destiny of lncRNA, thereby advancing the progression of CC, and have shown that decreasing m6A levels stabilises HOXC13-AS in CC cellular contexts (52).

ALKBH5 is another m6A demethylase, and although FTO belongs to the same ALKB family, the two “erasers” function differently because of their differential expression in different tissues (53, 54). ALKBH5 can not only reverse m6A methylation but also affect mRNA export and RNA metabolism. ALKBH5 is classified as a nonheme oxygenase that is typically found in the nucleus and is generally expressed at low levels in the cytoplasm.

ALKBH5 expression is significantly higher in OC tissues than in normal ovary, yet it is comparatively lower in OC cell lines than in cultured normal ovarian cells (55). Under hypoxia, the expression of ALKBH5 in breast cancer cells was up-regulated by hypoxia-inducible factor, which resulted in a decrease in the m6A modification level of NANOG mRNA, which in turn increased the NANOG protein level and promoted the enrichment of breast cancer stem cells (56). In addition, it has been found that high ALKBH5 expression in glioblastoma stem cells is associated with poor clinical outcome in glioblastoma patients (57).

### 2.3 Readers

The m6A “reader” proteins are composed of a group of YTH domain-containing proteins, which include YTHDF1, YTHDF2, and YTHDF3, as well as YTHDC1 and YTHDC2. Additionally, this category encompasses IGF2BP, a protein that binds the mRNAs of insulin-like growth factors, and the HNRNP family, which are involved in the processing and regulation of RNA within the cell nucleus (58, 59). With increasing m6A “reader” research, these proteins have been shown to potentially change the fate of modified mRNAs.

For example, after YTHDF1 attaches to the m6A-modified site, it brings in the EIF3A complex, which is a transcription initiation factor, to stimulate the commencement of translation. This process facilitates the aggregation of ribosomes into polyribosomes, thereby enhancing the efficiency of protein synthesis (60). The YTH domain family proteins YTHDF1 and YTHDF3 accelerate the translation of mRNAs by recruiting translation initiation factors, whereas YTHDF2 synergises with YTHDF3 and YTHDF1 to reduce mRNA stability, strengthen the decay and demethylation of their RNAs, and induces the degradation of m6A-modified target mRNAs (61, 62). YTHDF2, as the first m6A reading protein to be discovered, has generally been demonstrated to play an oncogene role in a variety of cancers. Li et al. showed a close correlation between increased YTHDF2 protein levels and OC tissue in the clinic (63). In prostate cancer, YTHDF2 expression was increased in cancer tissues compared with adjacent normal tissues (64).

YTHDC1 binds competitively to SRSF3 and SRSF10 to recognise methylated mRNAs and regulate the alternative splicing of mRNAs (65–67). Furthermore, YTHDC1 plays a pivotal role in the nuclear export of mRNA, facilitating its transfer to the cytoplasmic receptor NXF1, which is crucial for mRNA transport out of the nucleus (68). YTHDC2 binds to m6A, promotes the efficient translation of target genes, mediates RNA decay, and decreases mRNA stability. The m6A reader YTHDC2 is essential for accelerating translation via m6A (69).

HNRNPS family proteins include HNRNPC, HNRNPG, and HNRNPA2B1 (70). HNRNPA2B1 interacts with RNA and regulates the alternative splicing of genes modified by m6A. The secondary structure of m6A-modified mRNA changes, regulating gene expression and enabling it to bind HNRNPC (71). HNRNPA2B1 was identified as an m6A reading protein thanks to its ability to promote alternative splicing of exons with METTL3. Meanwhile, the binding of HNRNPA2B1 to DGCR8 protein provides it with the function to process pre-miRNAs (58).

The IGF2BP family, consisting of IGF2BP1, IGF2BP2, and IGF2BP3, is known to increase translation and engage in the regulation of cellular metabolism. These proteins recognise m6A modification sites on target mRNAs, thereby stabilising these mRNAs and modulating their translational efficiency (72, 73). Increased expression of IGF2BP family proteins has already been observed in previous cancer-related studies (74). It can enhance the stability and translation efficiency of specific target mRNA transcripts in cervical cancer cells by recognising and combining m6A modifications, thereby promoting proliferation, colony formation, migration, and invasion of tumour cells (59).

### 2.4 m6A in mRNA nucleation

Following a complex series of processing, mRNA molecules that complete maturation in the nucleus are transported into the cytoplasm for protein synthesis. Throughout this process, the m6A modification is crucial for the regulation of mRNA translation, as it generates specific spatial hindrances that influence the translational machinery. The mRNA molecule that has been modified by m6A is identified by the nuclear protein YTHDC1. Through interactions with splicing factors and the nuclear export adapter protein SRSF3, YTHDC1 ensures the efficient handover of the mRNA to the nuclear export receptor NXF1 (66). YTHDC1, in complex with SRSF3, mediates RNA-NXF1 binding to increase nuclear mRNA export (66). In addition, FMRP, a reader of m6A-modified RNA, plays an integral role in exportin 1 (XPO1)-mediated nuclear export (75, 76).

### 2.5 m6A in mRNA translation

The m6A modification increases translation efficiency through interactions with translation-related proteins. m6A-related translational regulation plays an essential role in a variety of cancers, as well as some normal physiological processes. METTL3 can recognise m6A located in the 5 ‘UTR and 3’ UTR, thereby accelerating translation (77). In addition, METTL3 interacts with eIF3, which further interacts with proteins associated with the cap structure of mRNAs to form mRNA loops.

METTL3 also affects ribosome biogenesis by regulating PES1 expression (78). Rui Su et al. reported that METTL16 can directly interact with eIF3a, eIF3b, and rRNA to promote translation and accelerate the assembly of translation initiation complexes in the cytoplasm (40). Interactions between YTHDFs and the translation machinery have been shown to increase translation efficiency. However, the precise mechanism by which YTHDF2 functions remains to be fully elucidated (79, 80). YTHDF1 facilitates the translation and elongation of the Snail mRNA by engaging with eEF2 and connects with ribosomes via cap-dependent initiation, interacting with eIF4G and eIF3. The effect of YTHDF1 is not limited to synergy with other translation factors, which can also increase eIF3C expression in an m6A-dependent manner (81).

YTHDF3 augments cap-independent translation during breast cancer brain metastasis by promoting the interaction of eIF3a with the m6A residue located in the 5’ UTR of the YTHDF3 mRNA (80). Although the eIF4 complex is required for the standard translation initiation process, YTHDF1 can initiate translation independently of the eIF4 complex by interacting with eIF3A and 3B (82). YTHDF3 works in concert with YTHDF1 to increase translation by interacting with the 40S ribosomal subunit (61). YTHDC2, a protein with RNA helicase activity, can improve the translation efficiency, but at the same time, it also leads to a decrease in mRNA abundance (83, 84).

### 2.6 m6A and mRNA stability

The m6A protein machinery is crucial for maintaining mRNA stability, regulating RNA metabolism, and sustaining the equilibrium of gene expression. Transcripts containing m6A-induced RNA decay via an m6A reader. The m6A modification exerts context-specific effects on mRNA stability, thereby regulating gene expression and contributing to cellular homeostasis (11, 85). Additional experimental findings indicate that the m6A modification exerts a dual influence on mRNA stability (86–88).

In particular, after YTHDF2 binds to m6A-modified mRNA, its degradation can proceed through at least two different pathways. On the one hand, when the binding domain of the temperature-sensitive protein HRSP12 and the cleavage domain for the endoribonuclease RNase P/MRP are located before and after the YTHDF2 binding domain, HRSP12 acts as an adaptor to link YTHDF2 with RNase P or MRP, thus promoting rapid degradation of RNA bound to YTHDF2 through the endoribonuclease route. On the other hand, YTHDF2 is capable of directly engaging the CCR4/NOT complex, which is associated with exosomes and processing bodies (P-bodies), to induce deadenylation, thereby initiating the breakdown of mRNAs that carry m6A modifications (89, 90). FTO can inhibit YTHDF2-mediated m6A-dependent RNA decay, thereby increasing MYC mRNA stability (91).

The YTHDF1 protein induces degradation by binding to the 3’-UTR m6A modification site of the MAT2A mRNA. YTHDF3 acts synergistically with YTHDF2 to induce the degradation of mRNA (61). Unlike YTHDF2, IGF2BP1/2/3 can increase mRNA stability by binding to RNA stabilisers. In addition, the FMRP and PRRC2A proteins enhance mRNA stabilisation in a manner dependent on the m6A modification by recognising m6A-modified mRNAs (86, 87). The intricacies of the mechanism regulating the m6A protein machinery have not been fully elucidated, necessitating future research to delve deeper into its precise mechanisms and identify additional RNA-binding proteins that may interact with this system.

## 3 m6A RNA methylation in female reproductive physiology

**Figure 2:**
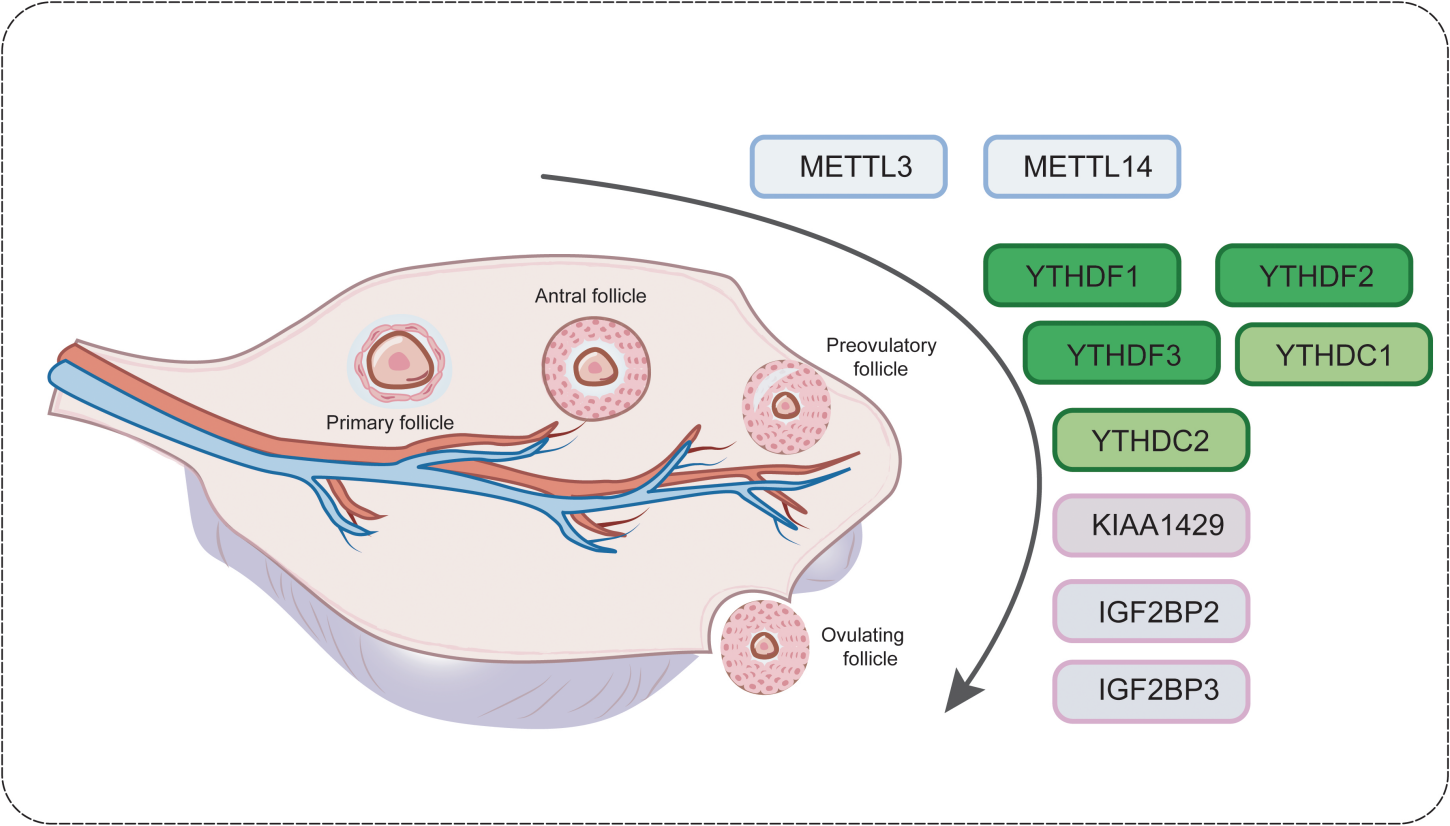
RNA methylation plays a pivotal role in early follicle development.

RNA methylation, especially m6A modification, plays a crucial role in oocyte maturation and early embryonic development, and plays an important regulatory role in gametogenesis and embryonic development. Oocyte maturation refers to the resumption of meiosis in oocytes in dominant follicles a few hours before ovulation and continued development from diplotene in the first meiotic prophase to metaphase in the second meiotic prophase (92) (**Figure 2**).

During oocyte maturation, it is often accompanied by a series of processes such as RNA storage, translation and degradation to maintain the homeostasis of relative changes in transcript dose in the transcriptome, so the precise regulation of intracellular RNA post-transcriptional levels appears to be particularly important during oocyte maturation, which has a very important role for egg quality, fertilisation process and early embryonic development (93). For example, Qi et al. showed that hypermethylated mRNAs were mainly enriched in progesterone-mediated oocyte maturation and cell cycle pathways, implying that m6A modification is involved in RNA translational regulation during oocyte maturation (94). Ivanova et al. further showed that YTHDF2 precisely regulates m6A modification-mediated mRNA degradation during oocyte maturation (95). In addition, the importance of METTL3 in oocyte maturation was highlighted by the study of Xia et al. by the arrest of oocyte maturation development in METTL3 mutants (96).

Embryonic development refers to the process from zygote development to embryo detachment from the oolemma. In early embryonic development, m6A modification similarly plays a critical role. Wu et al. revealed the important and unique significance of m6A modification during MZT through egg and embryo samples deficient in m6A modification (97). In addition, m6A modification in combination with miRNAs regulates the process of embryonic development, and a study by Hao et al. (98). MiR-670 was shown to play a key role in embryonic development by regulating IGF2BP1 expression through m6A modification. In summary, m6A modification plays an integral role in oocyte maturation and early embryonic development by precisely regulating RNA metabolism and stability, and its dynamic changes are essential for maintaining normal reproductive and developmental processes.

### 3.1 Germ cell development

Many studies have underscored the pivotal function of the m6A modification in governing cellular activities within the female reproductive system, including proliferation, differentiation, metabolic processes, and the cell cycle. Recent findings highlight the importance of the m6A modification during egg development. This conclusion is rooted in the observed significant differential clustering of m6A-modified genes in key signalling pathways involved in steroid synthesis, granulosa cell growth, and follicular maturation (99).

During the stages of egg maturation and late embryonic development, the RNA methylation process is involved in the fine regulation of RNA translation (94). Because transcriptional activity is not active during the initial stages of embryonic development, tight control of the translation process is essential for oocyte maturation (100). If the methylation level of maternal RNA is too high, it may interfere with the normal translation mechanisms and reduce the synthesis of proteins required for embryonic development and oocytes. In addition, recent findings support the role of m6A-related proteins in regulating ovulation processes, particularly METTL3, 14, YTHDC1/2, YTHDF1/2/3, and KIAA1429.

The absence of METTL3 function can lead to the blockade of the gamete maturation process, which in turn affects the reproductive capacity of individuals. This effect may be associated with a reduction in m6A modification levels, which may interfere with the normal expression of key genes involved in sex hormone synthesis and gonadotropin signalling pathways (101). When METTL3 activity is inhibited in oocytes, abnormal follicles form, which in turn affect the ovulatory process. In addition, the ability of METTL3 to regulate oocyte meiotic stability is enhanced by an m6A modification-dependent mechanism through cooperation with ITSN2, a protein that plays a restorative role during oocyte meiosis. In the presence of decreased METTL3 expression, females are unable to produce mature MII oocytes, and GV oocytes are smaller than normal in number and size, suggesting that METTL3 acts as an m6A methyltransferase to promote oocyte development by maintaining the stability of maternal mRNAs during GV (20). Although a reduction in METTL3 does not affect meiotic resumption, oocytes lacking METTL3 are defective in the formation of the first polar body and spindle.

L-Ascorbic acid treatment significantly reduces METTL14 mRNA levels in porcine oocytes, which may enhance the meiotic maturation and developmental competence of these cells (102). These findings suggest that METTL14 may play a critical role in oocytes during ovulation.

Several studies have shown that YTH domain-containing proteins such as YTHDC1/2 and YTHDF1/2/3 are essential for the ovulatory process (103–107). The oocyte maturation process is significantly affected by the YTHDC1 protein, and the loss of YTHDC1 is lethal to the embryo. Specifically, deletion of the m6A-modified reader YTHDC1 results in extensive changes in the polyadenylation pattern of the 3’-UTR, which affects microRNAs as well as protein-binding sites that are quite abundant in the 3’-UTR. When YTHDC1 function is inhibited after birth, female mice suffer a loss of fertility and are unable to form secondary and antral follicles. Notably, YTHDC1 deficiency also results in defective RNA metabolism and particle accumulation in oocytes, and these changes significantly affect oocyte maturation and ovulation processes (103).

Deletion of the YTHDC2 gene did not affect mouse survival but caused male mice to lose fertility and female mice to have a reduced ovarian size (106). YTHDC2 increases the efficiency of the translation process, stabilises target proteins by recognising m6A modifications, and may be involved in regulating self-renewal mechanisms in female germline stem cells. In the testes of male mice, YTHDC2 helps mitigate reproductive toxicity and cell cycle arrest (108).

Diminishing the expression of the YTHDF1 gene has been shown to curtail the self-renewal capacity of female germline stem cells in mice. Compared with cells derived from Sando’s inbred mouse embryos resistant to thioguanine and valine, these female mouse germline stem cells presented significant differences in global m6A levels mediated by METTL3, ALKBH5, YTHDF1/2, and YTHDC1/2 (109). In contrast to cells originating from Sando’s inbred mouse embryos, which are resistant to thioguanine and valine, notable disparities in the overall m6A levels were observed among these female mouse germline stem cells, particularly with respect to the expression of METTL3, ALKBH5, YTHDF1/2, and YTHDC1/2 (81). The loss of YTHDF2, on the other hand, blocks the degradation of m6A-modified mRNAs, which in turn affects oocyte quality (110). When YTHDF2 and YTHDF3 are mutated at the same time, they impair the normal development of the female gonad (111). These findings support earlier findings that the m6A modification and its associated proteins and mechanisms remain consistent among regulators of gametogenesis (112).

KIAA1429, which is recognised as a novel enzyme associated with m6A methylation, plays a critical role in oogenesis. Studies have indicated that in the absence of KIAA1429, oocytes exhibit disrupted regulation of apoptosis and proliferation in granulosa cells during the early stages of folliculogenesis. Additionally, abnormalities in chromatin structure and RNA metabolism are observed, suggesting that KIAA1429 is essential for maintaining the normal developmental trajectory of oocytes (113). Several other m6A recognition proteins, such as IGF2BP2 and IGF2BP3, are essential for DNA repair and the stability of maternal mRNAs during meiosis during the maturation stage of eggs (63). While preserving mRNA stability, m6A may also be involved in oocyte maturation through various metabolic pathways. Currently, the significant role of the m6A modification in oogenesis is widely acknowledged; however, its potential as a therapeutic target for treating ovulatory disorders requires further in-depth investigation.

### 3.2 Embryo development

Many studies have shown that m6A-related proteins, including METTL3, METTL5, IGF2BP2, and IGF2BP3, are also involved in embryonic development. The absence of METTL3 has been noted to lead to increased levels of naïve pluripotency transcripts within mouse embryonic stem cells, an alteration that impedes the initiation of cell differentiation and their subsequent developmental progression (85). Similarly, studies have revealed the methylation of 18S rRNA by METTL5. When METTL5 was knocked down, mouse embryonic stem cells (mESCs) lost their pluripotency profile.

*In vitro*, although the loss of IGF2BP2 did not affect the process of egg formation, general decreases in transcriptional and translational activities were observed at the two-cell stage of embryonic development. IGF2BP2 significantly contributes to the advancement of embryonic development to the blastocyst phase and increases the overall quality of embryos through the modulation of IGF2 expression (114).

The IGF2BP3 protein has a strong affinity for maternal mRNA, preserving its stability by preventing its degradation during the initial phases of embryogenesis. In the absence of IGF2BP3, the degradation rate of maternal RNA is accelerated. IGF2BP3 deficiency does not negatively affect oocyte development, and these cells are still able to develop normally. Notably, cell division is impaired in cells with IGF2BP3 mutations, which may have an impact on other aspects of early embryonic development (115).

### 3.3 Foetal growth

In addition to its influence on female germ cell development and embryogenesis, the m6A demethylase FTO plays a significant role in foetal growth. Studies have shown that FTO activity may affect the process of foetal development by regulating the expression of genes associated with foetal growth through specific molecular mechanisms (116). In newborns with low birth weights, m6A modification levels in the 5’ untranslated region (UTR) are higher, whereas m6A modification levels near the stop codon region are relatively lower than those in normal weight children of the same age (117, 118).

In particular, the expression of the FTO protein and the levels of the m6A modification on mRNA were significantly increased in the placentas of pigs with low birth weights under conditions of maternal obesity. Furthermore, in these low-birth-weight foetuses, the expression patterns of key genes involved in lipid metabolism and angiogenesis differed from those in normal foetuses. Researchers have hypothesised that elevated m6A levels might be involved in the regulation of the expression of key genes within placentas associated with low birth weight (119).

### 3.4 m6A functions in the innate immune response

Innate immunity constitutes a fundamental barrier against pathogen invasion, and a series of innate defensive cells, such as mononuclear phagocytes (MFs), natural killer (NK) cells, and dendritic cells (DCs), play key roles at the maternal–foetal interface. These cells have the ability to rapidly recognise invasive microorganisms and heterologous RNAs, which in turn trigger defence mechanisms against nonself pathogens (120, 121).

m6A modifications and their associated proteins are pivotal mediators of innate immune responses. They modulate the innate immune system by controlling mechanisms underlying cellular recognition and responses to external pathogens, unmodified tRNAs, exogenous RNAs, and aberrant endogenous RNAs. Specific knockout of the METTL3 gene in dendritic cells blocks the maturation of these cells when they encounter lipopolysaccharide (LPS), impairing their phenotype and normal function (122). In the innate immune system, macrophages constitute another indispensable cell population, and an analysis of RNA-binding proteins using CRISPR screening techniques identified preferential targets of m6A methylases that control LPS-induced macrophage activation. METTL3-deficient macrophages produced small amounts of TNF-α upon LPS stimulation. However, the suppression of METTL3 in macrophages notably amplified the production of proinflammatory cytokines such as TNF-α, IL-6, and nitric oxide (NO) (123).

The specific depletion of the m6A methylase METTL14 in macrophages disrupts the differentiation of CD8^+^ T cells, which in turn weakens the ability of these cells to clear tumours (124). Natural killer (NK) cells are crucial for the defence against tumours and viral infections. As an m6A-binding protein, YTHDF2 expression is significantly increased when NK cells are activated by cytokines, tumour cells, or viruses. Elevated levels of YTHDF2 are implicated in modulating the tumour suppressor functions of NK cells and are linked to the final maturation stages of NK cells. This maturation is vital for governing the migration and function of NK cells, thereby influencing their capacity to exert antitumour and antiviral effects within the organism (125). However, the impacts of the m6A modification on the development and functions of macrophages and NK cells are not yet fully understood and require further research and scrutiny.

### 3.5 Maternal–foetal immune microenvironment

At the maternal–foetal interface, the equilibrium of the immune microenvironment is orchestrated by the dynamic interplay between placental and maternal cells, a balance that is subject to changes across the continuum of pregnancy. Placental trophoblasts serve as innate immune cells, engaging in communication with maternal cells to ensure that the embryo develops in an optimal immune environment conducive to its growth and well-being. Indeed, any dysfunction in placental cells or maternal decidual cells can precipitate pregnancy complications, with early pregnancy loss, including recurrent miscarriage, being a notable example. RNA methylation is crucial for modulating transcription across a spectrum of cellular processes. Many studies have underscored the pivotal function of m6A and its regulatory components throughout the process of embryo implantation. They play substantial roles in governing the behaviour of innate and adaptive immune cells, tuning immune responses, and shaping local and systemic immune contexts, which may subsequently affect the immune environment at the maternal–foetal junction. However, the role of the m6A modification at the maternal–foetal interface remains insufficiently explored. In this review, we delve into the critical functions of m6A during early embryonic development and assess current studies on the regulatory effects of m6A on immune cells and the tumour immune background. The m6A modification is believed to have the potential to promote embryonic development, bolster placentation, and aid in shaping the immune microenvironment at the maternal–foetal interface. A thorough comprehension of these key regulatory mechanisms may clarify the path for innovative therapeutic strategies that leverage RNA methylation, particularly during the early stages of pregnancy.

## 4 m6A modifications in female reproductive system neoplasms

**Figure 3:**
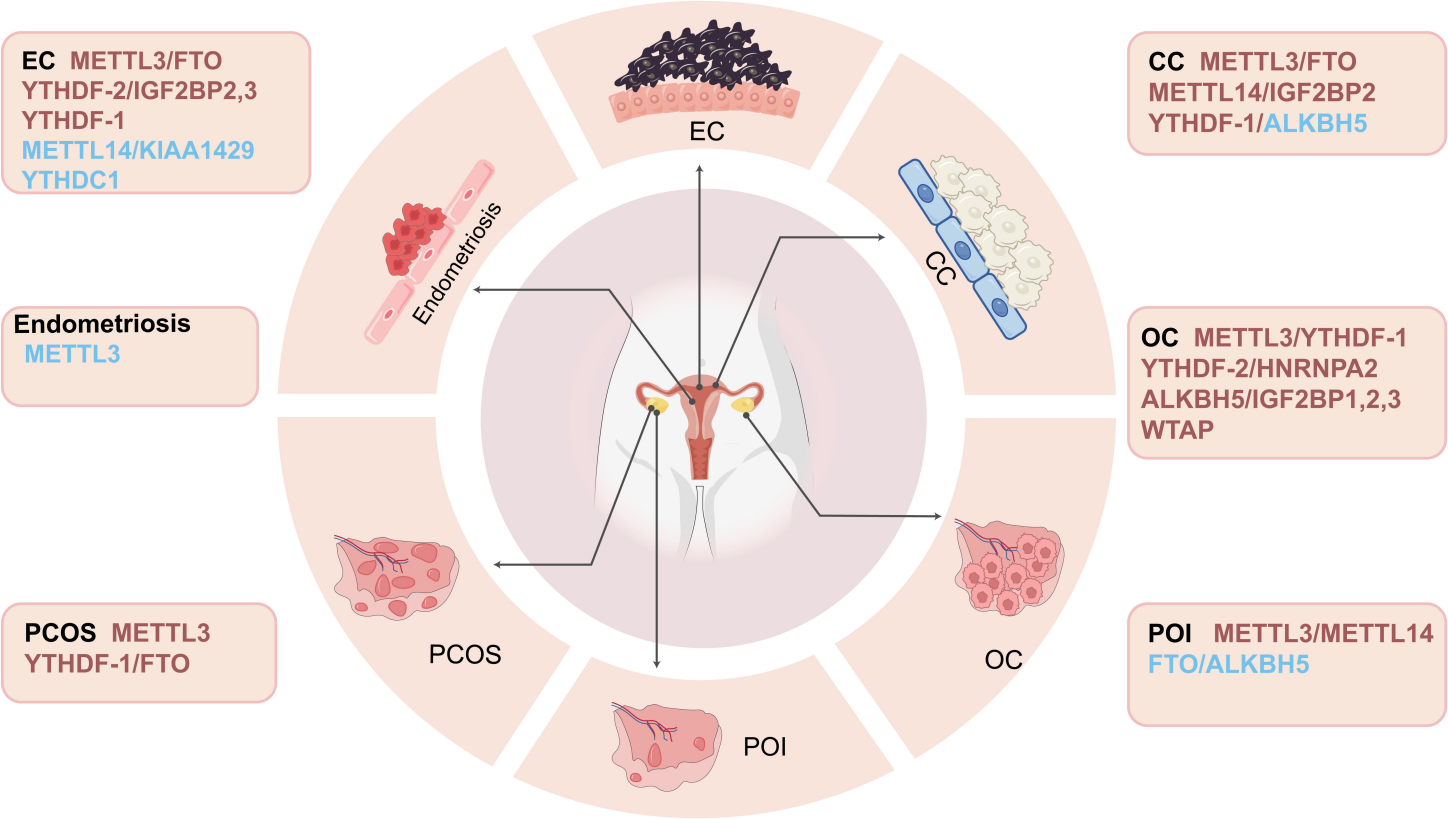
Illustrates the important role of m6A regulators in female reproductive disorders. These factors play a crucial role in the pathological changes of the female reproductive system, and they affect the pathogenesis of female reproductive disorders by regulating different molecular pathways and their corresponding targets (in which up-regulated targets are marked in red and down-regulated targets are marked in blue).

The m6A RNA modification is central to the regulation of gene expression and cellular function, with evidence showing that the dysregulation of m6A is associated with the pathogenesis of a range of diseases, including gynaecological disorders (**Figure 3**). In tumour cells, the transcriptional process of epithelial cells promotes carcinogenesis by regulating the expression levels of m6A modification-related enzymes, including “writers”, “readers”, and “erasers”. Specifically, up- or downregulation of the expression levels of these enzymes can affect the dynamic balance of m6A modifications, which in turn may drive tumour development (126–128). The m6A RNA modification is central to the pathogenesis of various cancers, with dysregulation of its modification patterns and underlying mechanisms contributing to the progression, growth, and spread of tumours by impacting the functional pathways of both coding and noncoding RNAs. Notably, specific m6A-modifying enzymes have been found to exert m6A-independent effects on certain malignancies, providing novel insights for cancer diagnostics and therapeutics.

Emerging research indicates that the m6A modification of mRNAs is intricately involved in reproductive processes, including oocyte maturation and the functionality of granulosa cells, through the regulation of RNA splicing, nuclear export, RNA degradation, and translation (129, 130). In addition, ROS-mediated oxidative stress leads to granulosa cell apoptosis and promotes follicular dysplasia, which can alter the m6A modification; however, how m6A participates in oxidative stress-induced ovarian cell apoptosis remains unclear (131, 132).

### 4.1 Preeclampsia

Placental trophoblasts play a key role in EMT in the initial stages of preeclampsia, a disease of placental origin. Upon the interference of multiple factors, the EMT process of trophoblasts may be abnormal, and this abnormality may lead to reduced invasion of trophoblasts into the uterine spiral artery, which in turn becomes a cause of preeclampsia (133). Numerous studies have revealed that trophoblasts in the placenta of patients with preeclampsia exhibit significantly increased levels of m6A methylation, METTL3, and HNRNPC1/C2 (134).

These chemical modifications have been shown to influence diverse biological attributes of placental trophoblasts. Some research teams have observed significant increases in m6A methylation and METTL3 expression levels in placental tissue from preeclampsia patients. Reducing METTL3 activity has been shown to significantly reduce m6A methylation levels in placental trophoblasts from individuals with preeclampsia, which in turn affects the expression of heterogeneous ribonucleoprotein complex members C1/C2 (hnRNPC1/C2); in addition, this process involves the maturation of miRNA 497-5p/145-5p (135). In the placenta of preeclampsia patients, the protein expression level of METTL14 has been shown to increase (136).

An in-depth exploration of the mechanism of action of METTL14 revealed that it was able to upregulate the expression of the FOXO3a gene through an m6A-dependent pathway, a process that not only promoted the autophagy and death of trophoblasts but also inhibited their proliferation and invasion (137). Compared with those in healthy placental tissue, no consensus is available on the changes in ALKBH5 and FTO levels in preeclamptic placentas. Some studies have suggested that the expression levels of these two proteins do not differ significantly in preeclamptic placentas, implying that their involvement in this condition might not be directly linked to their expression levels. Instead, it could be related to alternative regulatory mechanisms or functional alterations (134).

However, others have reported a significant increase in the expression level of ALKBH5 in placentas from preeclampsia patients, as well as in cells subjected to hypoxia or reoxidation (138). At the mechanistic level, the expression of the RNA demethylase ALKBH5 is notably elevated in placental tissues affected by preeclampsia. This increase in ALKBH5 expression decreases the stability of the PPARG mRNA, thereby disrupting its normal translation. Consequently, this process affects the demethylation of ALCAM by the histone demethylase KDM3B, causing a decrease in ALCAM expression. This chain of molecular events is implicated in the pathogenesis of preeclampsia (138).

Recent explorations of the pathomechanism of preeclampsia have focused mostly on the IGF2BP protein family, which is expressed at low levels in preeclamptic placental samples (139). IGF2BP3 contributes to increasing circRNA PAPPA2 stability by recognising and binding m6A modifications. In addition, the MNSFβ protein can directly interact with IGF2BP2 and enhance the invasion and migration of trophoblast cells (e.g., JEG-3 and HTR8/SVneo) (139).

A sequencing analysis of m6A-modified RNA in PE placentas revealed that circRNA m6A methylation levels were increased in preeclamptic placentas compared with normal placentas. Recent studies have shown that the YTHDC1 protein maintains the normal function of trophoblasts by accelerating the degradation of m6A-modified circMPP1 (140). Recent studies have shown that YTHDF3 expression levels increase in trophoblast cells under hypoxic conditions and that its inhibition of IGF1R gene expression by the m1A modification leads to decreased trophoblast invasion (141). Because hypoxia is involved in the pathogenesis of preeclampsia, YTHDF3 may be involved in the pathogenesis of preeclampsia.

### 4.2 Abortion

Spontaneous abortion represents one of the most prevalent complications encountered in pregnancy. Controlling the invasion of trophoblasts and reducing their atypical growth and division can reduce the impact on the health of the placenta *in utero*. Thus, trophoblasts are essential for the formation of the placenta. During pregnancy, dysfunction of trophoblast molecules may trigger serious pregnancy problems (142, 143). When human trophoblasts are exposed to BPDE, METTL14 expression levels are increased compared with those before exposure, which was also observed in the placental villous tissue of women with recurrent miscarriages.

Increased ALKBH5 levels in the placental villous tissue of those with recurrent miscarriages may explain the noted decrease in global m6A modification levels. ALKBH5 may affect trophoblast invasiveness by reducing CYR61 mRNA stability through an m6A-dependent mechanism (144). The expression levels of FTO, an RNA demethylase, are decreased in women who experienced a spontaneous abortion. These findings suggest that the abnormal accumulation of m6A at the maternal–foetal interface may be a factor contributing to spontaneous abortion (145).

The m6A modification may increase IGFBP3 transcript levels, which in turn may increase MMP2 activity while decreasing the expression of its specific inhibitor tissue inhibitor TIMP-1. Eventually, spontaneous abortion occurs (146).

### 4.3 Endometriosis

Endometriosis, an oestrogen-independent condition, is characterised by the ectopic occurrence of endometrial-like tissues outside the uterine cavity. Patients typically present with intense pelvic pain and infertility, significantly impairing their daily life and productivity (147–149). Many studies have shown that the pathogenesis of endometriosis may be related to m6A, and some of these regulators, including METTL3, YTHDF2, YTHDF3, HNRNPC, HNRNPA2B1, and FTO, are significantly dysregulated in the ectopic endometrium (150).

Histopathological assessments of endometriosis patients have indicated a notably lower m6A modification level in endometrial tissues than in healthy control tissues, a finding that may be correlated with the decreased expression of METTL3. This finding suggests that decreased METTL3 expression enhances the migration and invasiveness of human endometrial stromal cells (HESCs) by inhibiting the DGCR8-mediated transformation of pri-miRNA126 to mature miRNA126, which in turn accelerates endometriosis development. On the other hand, METTL3 overexpression has the opposite effects, which may provide ideas for further studies of endometriosis (151).

HNRNPA2B1 and HNRNPC are involved in the regulation of immune responses, and their abnormal expression is closely related to the pathogenesis of endometriosis. Therefore, they can be used as biomarkers of endometriosis to assist with diagnosis and treatment (150). Although research on the role of the mechanisms of m6A-related proteins in endometriosis is still limited, the existing studies have highlighted their substantial effects on this condition. These insights are anticipated to yield potential biomarkers and therapeutic targets, paving the way for the improved clinical management of endometriosis.

### 4.4 Adenomyosis

Adenomyosis is a relatively common uterine disease of the female reproductive system that is characterised by the excessive proliferation of abnormally accumulated epithelial cells and stromal fibroblasts in the endometrium, causing hypertrophy and hyperplasia of adjacent smooth muscle cells (152–154). Currently, the pathophysiological mechanisms underlying adenomyosis are not fully understood, and effective biomarkers and treatment options are lacking.

Zhai et al. reported that the m6A protein machinery is involved in the development of adenomyosis. Notably, regulators of m6A RNA methylation, including METTL3, ZC3H13, and FTO, are expressed at reduced levels in the myometrium of individuals with adenomyosis, consequently affecting global m6A levels (155). A correlation between the significant upregulation of IGF1 and DDT gene expression levels and a reduction in METTL3 expression levels has already been validated. A number of additional potential target genes, such as cadherin 3 (CDH3), placenta-specific protein 8 (PLAC8), and sodium channel β-subunit 4 (SCN4B), have been shown to significantly contribute to processes such as cell adhesion, muscle contraction, and immune responses within the myometrium of patients with adenomyosis. Previous studies have indicated that these genes participate in the regulation of epithelial cell proliferation and migration.

Therefore, the pathogenesis of adenomyosis may be related to the cellular processes associated with these genes (155, 156). These studies provide new perspectives and possible therapeutic directions for the diagnosis and treatment of adenomyosis. However, these experimental results have not been validated in animal models or in clinical trials. Therefore, future studies need to further explore its mechanism of action and discover its diagnostic markers and therapeutic targets.

### 4.5 Polycystic ovary syndrome

PCOS is a highly complex disease with an uncertain aetiology in the metabolic and endocrine fields. Patients often show clinical associations with obesity, insulin resistance (IR), and cardiovascular disease (157, 158). In addition, obesity not only exacerbates metabolic disorders but also adversely impacts oocyte quality and may interfere with endometrial receptivity, factors that collectively influence fertility in women of reproductive age (159, 160). Recent studies have revealed elevated levels of the m6A modification in luteinised granulosa cells (GCs) from patients with PCOS. The loss of the m6A modification of the FOXO3 mRNA was also observed in luteinised granulosa cells from PCOS patients. By selectively knocking down m6A methyltransferases or demethylases, researchers have observed a change in FOXO3 expression in luteinised GCs from controls but not in those from PCOS patients. This series of findings suggests the possible inhibition of m6A-mediated transcriptional regulation of the FOXO3 gene in luteinised GCs from PCOS patients. FOXO3 is a key target of METTL3, which modifies the FOXO3 3’-UTR-untranslated region, increasing its stability and activating its autophagy-related pathway through a YTHDF1-dependent mechanism (161). Interestingly, autophagy is prevalent in ovarian tissue from patients with polycystic ovary syndrome (PCOS). In luteinised granulosa cells from PCOS patients, FTO facilitates m6A demethylation by downregulating the m6A modification of the FLOT2 mRNA, a mechanism that correlates with insulin resistance and a phenotype characterised by an elevated proliferation rate and diminished apoptosis in these cells (162). The above studies may reveal the potential pathogenesis of and future treatment ideas for PCOS, and more studies on PCOS are needed.

### 4.6 Primary ovarian insufficiency

Primary ovarian insufficiency (POI), also known as premature ovarian failure, is a clinical condition characterised by persistent amenorrhea, elevated follicle-stimulating hormone (FSH) levels, and reduced oestrogen levels in women under the age of 40. The exact pathomechanism of this condition has not been fully elucidated, but chemotherapeutic agents, particularly alkylating agents such as cyclophosphamide (CTX), may lead to impaired ovarian function by affecting m6A RNA methylation levels (163–165).

Specifically, CTX has been reported to increase m6A methylation levels and the expression of the methyltransferases METTL3 and METTL14 in a concentration- and time-dependent manner while decreasing the expression levels of the demethylase FTO and the effector protein YTHDC1 in human granulosa cells and mouse models (166). In a previous study, an increase in m6A levels and a decrease in FTO expression were observed in POI patients. In addition, the incidence of apoptosis increases, whereas the proliferation decreases in granulosa cells in which FTO expression is knocked down (132).

Increased levels of the m6A modification of mRNAs were observed in male mice deficient in the ALKBH5 gene. These mice presented a notable decrease in the sperm count and abnormal sperm morphology, along with altered sperm motility. In addition, mice lacking ALKBH5 may undergo apoptosis during the metaphase and coarse stages of spermatocytes, which is accompanied by disturbances in spermatogenesis. Given that ALKBH5 is expressed in the reproductive tissues of both males and females, it is inferred to play a significant role in germ cell development (167). Dysregulated levels of m6A modifiers could impact oocyte maturation by influencing cell division processes during meiosis.

### 4.7 Endometrial cancer

EC, which is prevalent in developed countries, typically arises from the endometrial epithelium and affects perimenopausal and postmenopausal women. Its diagnosis and treatment often necessitate invasive evaluations and surgical procedures (168, 169). The standard therapeutic approaches for endometrial cancer (EC) include surgical intervention, radiation therapy, hormone treatment, and targeted therapy (170). The prognosis is usually optimistic if the disease is diagnosed early and treated with surgery (171). However, patients with advanced or poorly differentiated EC face a high risk of recurrence and a poor prognosis even after multimodal treatment because of its invasive metastatic properties (172). Thus, exploring and investigating biomarkers and their molecular mechanisms in EC are essential to improve patient outcomes.

In EC, abnormal expression or dysfunction of m6A regulatory proteins may lead to the abnormal regulation of tumour-related genes, which leads to the development and progression of EC. In a 2018 study, mutations in METTL14 or decreased expression of METTL3 resulted in decreased m6A methylation levels in endometrial tumours, and this decrease promoted the proliferation and tumour formation of EC cells by activating the AKT signalling pathway and its regulators PHLPP2 and mTORC2 (27). Another study in the same year revealed that specific knockdown of the m6A methylase METTL14 in macrophages impaired the antitumour activity of CD8+ T cells, a phenomenon that was also observed in colorectal cancer, providing a new perspective for studies of the immune microenvironment in EC (173).

IGF2BP1 increases PEG10 mRNA expression by recognising the m6A modification site in the PEG10 mRNA, thereby promoting the proliferation of EC cells (174). In addition, the expression level of PADI2 is increased in EC and increases the expression of IGF2BP1, which in turn binds to the m6A locus in the 3’ untranslated region of the SOX2 mRNA and increases its stability, leading to the malignant progression of EC (175, 176).

IGF2BP1 expression is increased in EC, and this high level of expression is strongly associated with a poor patient prognosis (177). IGF2BP1 is also involved in arginine guanidinium methylation in EC, and dysregulation of IGF2BP1 expression mediated by the PADI2/MEK1/ERK signalling pathway leads to the upregulation of SOX2 expression and subsequently promotes the malignant phenotype of EC cells (176). High expression levels of IGF2BP1 are also strongly associated with a poor prognosis for EC patients. These findings reveal multiple roles for IGF2BP1 in EC development, including promoting cell proliferation, regulating the tumour cell cycle, and promoting tumour progression.

In EC, a decrease in the level of m6A-modified RNA leads to a significant decrease in the expression level of KIAA1429 (176). The expression of the KIAA1429 gene is closely associated with nuclear metabolism. However, in liver cancer, KIAA1429 is known to promote liver tumour progression by regulating the posttranscriptional modification of GATA binding protein 3 (GATA3), a highly conserved essential transcription factor that is widely expressed in a variety of tissues, in an N6-methyladenosine-dependent manner (178). The specific mechanism of action of KIAA1429 in EC remains to be further elucidated.

In EC, ALKBH5 expression is notably elevated under hypoxic conditions, facilitating SOX2 transcription via mRNA demethylation. This process sustains the stem-like features and tumourigenic potential of endometrial cancer stem cells (ECSCs), thereby driving the aggressive progression of EC (179). Consistent observations from studies in 2020 have indicated that ALKBH5 levels are elevated in EC tissues. By diminishing the m6A methylation of IGF1R, ALKBH5 increases the stability of the IGF1R mRNA, stimulates IGF1R translation, and activates the IGF1R signalling cascade, consequently fuelling the proliferation and invasive capabilities of EC cells (180, 181).

In EC tissues, a marked increase in the expression of FTO has been observed. Increased levels of FTO can facilitate the demethylation of the m6A mark in the 3’ untranslated region (3’ UTR) of the HOXB13 mRNA, a modification that prevents YTHDF2 from recognising the m6A site. This demethylation leads to the activation of the WNT signalling pathway and subsequent regulation of downstream protein expression, ultimately promoting tumour metastasis and invasion (182, 183).

In EC, the suppression of YTHDC1 is associated with increased proliferation and invasion of cancer cells (184). However, YTHDF2 exerts the opposite effect and is capable of curbing the proliferation and invasive behaviour of EC cell lines. Specifically, the m6A reader YTHDF2 can identify and bind to the methylation site on the target transcript insulin receptor substrate 1 (IRS1), a mechanism that promotes the degradation of the IRS1 mRNA, thereby suppressing IRS1 expression and consequently impeding the IRS1/AKT signalling pathway to ultimately curb the tumourigenic potential of EC (185).

WTAP, which is recognised as an essential m6A methyltransferase, facilitates the addition of m6A modifications to mRNAs, particularly within the 3’ untranslated region (3’-UTR) of transcripts such as CAV-1. A decrease in CAV-1 expression triggers the activation of the NF-κB signalling cascade, thereby significantly contributing to the progression of EC (186). Based on the aforementioned research, a distinct correlation exists between m6A regulators and their integral roles in both the clinical treatment and diagnosis of various diseases.

### 4.8 Cervical cancer

Cervical cancer (CC) is indeed one of the most prevalent gynaecological tumours globally, with a well-established link to persistent human papillomavirus (HPV) infection, which is identified as a significant risk factor for its development. In summary, the TME significantly influences the initiation, progression, and outcomes of CC (187). Although the widespread use of HPV vaccines has reduced the incidence of CC to some extent, because the early symptoms of CC are not obvious, early detection and treatment are more difficult in clinical practice, with a five-year survival rate of less than 20% and a poor prognosis for patients with CC recurrence (188, 189). Hence, understanding the molecular mechanisms of CC is crucial for identifying novel therapeutic targets and enhancing clinical treatment approaches.

The m6A modification is instrumental in the progression of CC, with METTL3, in particular, shown to increase the proliferation and invasiveness of CC cells (190). Wang et al. observed that METTL3 levels are substantially elevated in CC tissues and cell lines, closely correlating with lymph node metastasis and unfavourable patient outcomes (191). In terms of the molecular mechanisms, METTL3 can target the 3’-UTR of the hexokinase 2 (HK2) mRNA and increase the stability of HK2 by recruiting YTHDF1, thereby promoting the Warburg effect in CC (191).

Furthermore, METTL3 facilitates the m6A modification of pyruvate dehydrogenase kinase 4 (PDK4), increases PDK4 mRNA translation, stimulates cancer cell glycolysis, and subsequently promotes the growth and progression of CC (192). Recent research has revealed an innovative pathway through which METTL3 enhances the invasiveness of CC by suppressing miR-193b activity, thereby upregulating “CCND1” expression and accelerating CC progression and severity (193). An earlier study revealed that elevated METTL3 levels in CC tissues were positively correlated with the presence of CD33^+^ cells within the tumour and with the clinical outcomes of patients (194). Elevated levels of METTL3 in CC tissues markedly increase the expression and stability of FOXD2-AS1, thereby further promoting the malignancy of CC (195).

METTL14 stimulates the expression of the tumour development and progression-associated protein seven in absentia homologue 2 (Siah2). The suppression of Siah2 hinders the proliferation and cytotoxic capabilities of T cells, as it sustains the expression of PD-L1 on tumour cells. Moreover, an examination of samples from patients subjected to anti-PD-1 immunotherapy indicated that tumours exhibiting reduced Siah2 levels responded more positively to the treatment (196).

FTO is highly expressed in human CC tissues and is closely associated with disease progression and patient prognosis (51). At the molecular level, FTO engages directly with the E2F1 and Myc mRNAs. The suppression of FTO markedly impacts the translation of these key oncogenes, subsequently curbing the proliferation and migration of CC cells (51).

In addition, the expression of GAS5-AS1 was decreased in CC tissues, and GAS5-AS1 combined with ALKBH5 and reduced the m6A modification of GAS5, which increased its stability, inhibited the proliferation and metastasis of CC cells, and affected the prognosis of patients (197). However, different investigators have different views on the relationship between FTO and CC, and some studies have revealed how HOXC13-AS promotes the proliferation, invasion, and EMT of CC cells by increasing the level of FZD6 and activating the Wnt/β-catenin signalling pathway (198).

Zhou et al. discovered that FTO modulates the expression of β-catenin by reducing m6A levels in the β-catenin mRNA, which in turn increases the chemoresistance of cancer cells both *in vivo* and *in vitro* (199). These findings reveal the multiple roles of FTO in CC development and highlight its potential as a novel therapeutic strategy and a critical target for prognostic evaluations.

YTHDF1 promotes translation of the RANBP2 protein through an m6A-dependent mechanism but does not alter the expression level of the RANBP2 mRNA, which in turn enhances the growth and invasion of CC cells. It is significantly correlated with unfavourable outcomes of CC (197).

Research indicates that the expression of the circRNA circARHGAP12 is increased in CC tissues, where it stabilises the FOXM1 mRNA through its interaction with IGF2BP2 in an m6A-dependent manner, contributing to the malignancy of CC (200). A separate study revealed that the long noncoding RNA KCNMB2-AS1 is markedly overexpressed in CC and is correlated with an unfavourable prognosis for patients. IGF2BP3 can identify and bind to the m6A-modified site on KCNMB2-AS1, increasing its stability and thus facilitating the progression of CC (201).

In addition, the IGF2BP3 and YTHDF1/eEF-2 complexes increase PDK4 mRNA stability through the m6A modification system, a process that promotes the glycolytic process of cancer cells and accelerates CC development (192).

### 4.9 Ovarian cancer

OC is a common cause of death in women with gynaecologic cancer, and its five-year survival rate is between approximately 30% and 40% (202, 203). Because early symptoms are not significant, many patients are already in an advanced metastatic state at the time of diagnosis, and the tumour has also spread to adjacent tissues (204).

The presence of metastatic tumours is a major cause of high mortality in OC patients. Although most patients’ symptoms can be significantly relieved by appropriate treatment at an early stage, approximately 70% of patients with advanced disease experience relapse (205). The cure rate and survival rate for patients with advanced OC have not improved significantly in recent years. This lack of improvement is partly attributed to the absence of effective early diagnostic screening and detection methods, coupled with high rates of tumour metastasis and recurrence (206). Consequently, a thorough investigation into the high-risk factors and the precise molecular mechanisms underlying the progression of OC is crucial for facilitating early intervention and treatment, thereby increasing patient survival rates.

Proteins associated with the m6A modification are instrumental in the initiation and progression of OC (207). Recent studies have shown that METTL3 plays a key role in the TME, and its increased expression in OC tissues is significantly correlated with the clinical characteristics of tumours. Luo et al. reported that the m6A modification can affect antigen presentation processes in the immune system and plays a critical role in cell infiltration in the TME of OC (208).

Hua and colleagues showed that stable overexpression of METTL3 under *in vitro* experimental conditions significantly enhanced the ability of nude mouse cells to proliferate, form tumours, migrate, invade, and form tumours (207). In contrast, in cell lines in which METTL3 expression was downregulated by a short hairpin RNA (shRNA), its function in cancer was effectively inhibited. Further mechanistic analysis revealed that METTL3 increases AXL mRNA expression, a process that accelerates the EMT, which in turn promotes the proliferation, tumour formation, migration, and invasion of OC cells.

The proportion of apoptotic cells increases when METTL3 expression is suppressed in OC cells, a phenomenon that may stem from the activation of mitochondria-mediated apoptotic pathways. A study by Liang et al. revealed that METTL3 may also play an active role in OC progression by increasing the phosphorylation of AKT and the expression level of Cyclin D1. In addition, METTL3 expression was significantly increased in OC tissues compared with other tumour tissues.

However, the expression levels of METTL3 and m6A are significantly increased in epithelial OC, and the degree of malignancy is high (108). Therefore, METTL3 may be used as a specific indicator of epithelial OC in the future.

YTH domain family proteins, through the chemical modification of m6A, can affect the development of OC at different levels. YTHDF1 and hnRNPA2 promote the development and metastasis of OC by increasing the expression levels of EIF3C and Lin28B, respectively, and are associated with a poor prognosis (81, 209).

The binding of the YTHDF1 protein to m6A-modified TRIM29 promotes the translation of TRIM29 in cisplatin-resistant OC cells, enhances the cancer stem cell characteristics in cisplatin-resistant OC cells, and then promotes the development of malignant tumours. These findings suggest that it may become a potential new target for OC treatment (109).

YTHDF2 increases proliferation and migration and inhibits apoptosis in epithelial OC cells through the YTHDF2/m6A regulatory axis while downregulating global mRNA m6A levels (63). YTHDF2 is a new substrate of the FBW7 enzyme, and the expression level of FBW7 is decreased in OC tissues. This decrease is closely related to a poor prognosis and a decrease in the m6A modification level. Further studies revealed that FBW7 triggers the proteasomal degradation of YTHDF2, thereby counteracting its tumour-promoting effect (210).

Recent studies on the tumour suppressor protein IFFO1 have revealed how METTL3 and the YTHDF2 axis regulate the stability of the IFFO1 mRNA through an m6A-dependent mechanism, which in turn inhibits the invasive ability of OC cells and reduces their drug resistance (211). When METTL14 is overexpressed, it effectively inhibits granulosa cell proliferation by inactivating PI3K/AKT/mTOR signalling. WTAP can regulate the proliferation and invasion of high-grade serous OC (HGSOC) cells by activating the AKT signalling pathway (212). Reducing the expression levels of METTL14 and WTAP has limited effects on the tumour development and behaviour of endometrioid epithelial OC cells cultured *in vitro* (108).

WTAP might stimulate the growth and mobility of high-grade serous ovarian cancer (HGSOC) cells and impede their apoptosis by triggering the MAPK and AKT signalling pathways. Furthermore, the long noncoding RNA UBA6-AS1 is able to recruit the RBM15 protein, which in turn increases the m6A methylation of the UBA6 mRNA, leading to the inhibition of UBA6 expression. This process is associated with the increased proliferation, migration, and invasion of ovarian cancer (OC) cells (213).

Zhang et al. discovered that FTO expression was elevated in OC tissues, where it increased OC cell viability and autophagy by stimulating AKT phosphorylation, thereby inhibiting apoptosis in cancer cells (214). Unlike previous studies in which FTO was used to promote tumourigenesis, a study focused on OC revealed that FTO expression levels are decreased in this type of cancer and inhibit tumourigenesis *in vivo* by affecting cAMP signalling pathways and blocking the self-renewal process of OC stem cells (215).

In epithelial ovarian cancer, ALKBH5 expression is notably elevated, and its suppression reduces the activity of the EGFR/PI3K/AKT signalling pathway (216). This process leads to an increase in autophagy signalling and the suppression of OC cell proliferation and invasion. Concurrently, research has indicated that ALKBH5 is more abundantly expressed in OC tissues, with reduced expression in OC cell lines, mirroring the expression pattern of TLR4. The activation of the m6A modification by TLR4 bolstered the NF-κB pathway, consequently increasing ALKBH5 expression levels (217).

Elevated expression levels of ALKBH5 in OC promote tumour cell proliferation and migration and inhibit the autophagy process by increasing BCL-2 mRNA stability, which in turn activates the EGFR-PIK3CA-AKT-mTOR signalling pathway (218). ALKBH5 overexpression is associated with OC with lymph node metastasis and may promote lymphangiogenesis and the lymph node metastasis of ovarian tumours by affecting the m6A modification and the ITGB1/FAK signalling pathway (219). ALKBH5 forms a regulatory loop with HOXA10 that can activate the JAK2/STAT3 signalling pathway by regulating the m6A demethylation of JAK2, a process that causes epithelial OC (EOC) to become resistant to cisplatin drugs (217).

IGF2BP1 increases SRF expression in an m6A-dependent manner by preventing SRF mRNA degradation by miRNAs and sustaining PDLIM7 and FOXK1 levels, thereby promoting tumour cell proliferation and invasion in OC (220). In high-grade serous OC (HGSOC), which presents mesenchymal features, the expression level of IGF2BP1 increases significantly, promoting the invasive growth of OC cells (221). In the same year, another study revealed that increased IGF2BP2 protein expression promoted the proliferation and invasion of OC cells by binding to circ0000745 and increasing its stability (222). IGF2BP3 is an RNA-binding protein that is upregulated in ovarian clear cell carcinoma (OCCC) and promotes cancer cell proliferation and tumour formation (216).

In summary, METTL3/14, YTHDF1/2, WTAP, FTO, ALKBH5, and IGF2BP1/2/3 play roles in promoting or inhibiting OC, and these factors are expected to be tools for the diagnosis and treatment of OC. However, many types of OC, including high-grade serous OC, endometrioid OC, and clear cell OC, have been identified. Whether the function of m6A is consistent in multiple OCs requires further investigation.

## 5 Conclusions and perspectives

Despite significant advancements in the field, considerable challenges remain in thoroughly understanding the role of m6A RNA methylation in the context of infertility and various reproductive diseases. This complex epigenetic modification is known to play a crucial role in regulating gene expression, but its specific mechanisms and implications in reproductive health issues have not yet been fully elucidated. Researchers continue to encounter difficulties in deciphering the intricate interplay between m6A modifications and the molecular pathways that contribute to fertility and the development of reproductive disorders. The heterogeneity of patient populations, the complexity of the female reproductive system, and the dynamic nature of RNA methylation patterns all contribute to ongoing challenges in this area of study.

First, the role of m6A modification in gynaecological tumours is currently based primarily on bioinformatics analyses and animal model research, with relatively few clinical trials. With the identification of proteins associated with the m6A modification, our understanding of the underlying mechanisms and signalling pathways has significantly increased. Despite the recognition that the m6A modification can either stimulate or suppress the progression of gynaecological cancers, a comprehensive grasp of the intricate processes driving oncogenesis is still lacking. Accordingly, more clinical trials are needed to elucidate these mechanisms, facilitating the development of more potent therapeutic strategies in the future.

Second, some m6A regulators may be associated with gynaecological diseases that have yet to be explored. The intricate operational mechanisms of m6A-related proteins within a range of prevalent gynaecological disorders are not yet fully understood, highlighting the need for additional investigations into how m6A influences multiple pathways that govern gene expression. Therefore, more in-depth studies are necessary to identify more m6A regulators associated with gynaecological diseases.

Third, reports on m6A-specific modification sites are currently limited, which limits their potential for clinical application. Although m6A RNA methylation is thought to influence other molecules, our understanding of its molecular mechanisms and cellular effects on gynaecologic reproductive system diseases is not comprehensive. Furthermore, the irregular expression patterns of m6A-modified regulatory molecules in gynaecological malignancies remain unclear. Enhancing the sensitivity and specificity of pertinent biomarkers is essential for advancing the clinical application of m6A regulators in diagnosis and therapeutics.

Fourth, research indicates that modulating m6A levels and the protein machinery could target specific reproductive diseases; however, clinical validation regarding safety, efficacy, and potential adverse effects, particularly on a large scale, is still pending. Currently, the m6A modification remains challenging despite advances in gynaecologic oncology research.

Fifth, no consensus is available on whether m6A modifications differ in their impacts on different tumour subtypes. More in-depth studies are needed to obtain answers for this question. The regulatory mechanisms associated with the m6A modification have been exhaustively explored in many types and different pathological stages of gynaecological malignancies.

In conclusion, the study of the m6A modification has emerged as a focal point of scientific inquiry, garnering significant attention for its potential implications across various fields of research. It is instrumental throughout the metabolic journey of mRNA, from its synthesis in the nucleus to its translation and ultimate degradation within the cytoplasm, thereby determining the ultimate fate of the mRNA. Relative to other relevant reviews, this manuscript provides an exhaustive examination of the relationship between the m6A modification and the physiological and pathological aspects of the female reproductive system. It encompasses a wide range of conditions, from germ cell genesis and embryonic development to benign and malignant diseases affecting female reproductive organs. The hope is that the m6A modification will become a pivotal factor in diagnosing and treating gynaecological conditions in the future.

## References

[1] Dwivedi SKD Rao G Dey A Mukherjee P Wren JD Bhattacharya R. Small non-coding-rna in gynecological Malignancies. Cancers (Basel). (2021) 13:1085. doi: 10.3390/cancers13051085 33802524 PMC7961667

[2] Schäfer KP. Rna synthesis and processing reactions in a subcellular system from mouse L cells. Hoppe Seylers Z Physiol Chem. (1982) 363:33–43. doi: 10.1515/bchm2.1982.363.1.33 6174404

[3] Roignant JY Soller M. M(6)a in mrna: an ancient mechanism for fine-tuning gene expression. Trends Genet. (2017) 33:380–90. doi: 10.1016/j.tig.2017.04.003 28499622

[4] Lipshitz HD Claycomb JM Smibert CA. Post-transcriptional regulation of gene expression. Methods. (2017) 126:1–2. doi: 10.1016/j.ymeth.2017.08.007 28867174

[5] Gilbert WV Bell TA Schaening C. Messenger rna modifications form distribution and function. SIGNALS IN RNA. (2016) 352:1408–12. doi: 10.1126/science.aad8711 PMC5094196 27313037

[6] Sun X Lu J Li H Huang B. The role of M(6)a on female reproduction and fertility: from gonad development to ovarian aging. Front Cell Dev Biol. (2022) 10:884295. doi: 10.3389/fcell.2022.884295 35712673 PMC9197073

[7] Huang W Chen TQ Fang K Zeng ZC Ye H Chen YQ. N6-methyladenosine methyltransferases: functions, regulation, and clinical potential. J Hematol Oncol. (2021) 14:117. doi: 10.1186/s13045-021-01129-8 34315512 PMC8313886

[8] Ma S Chen C Ji X Liu J Zhou Q Wang G. The interplay between M6a rna methylation and noncoding rna in cancer. J Hematol Oncol. (2019) 12:121. doi: 10.1186/s13045-019-0805-7 31757221 PMC6874823

[9] Ping X-L Sun B-F Wang L Xiao W Yang X Wang W-J. Mammalian wtap is a regulatory subunit of the rna N6-methyladenosine methyltransferase. Cell Res. (2014) 24:177–89. doi: 10.1038/cr.2014.3 PMC3915904 24407421

[10] Yue Y Liu J Cui X Cao J Luo G Zhang Z. Virma mediates preferential M(6)a mrna methylation in 3'utr and near stop codon and associates with alternative polyadenylation. Cell Discov. (2018) 4:10. doi: 10.1038/s41421-018-0019-0 29507755 PMC5826926

[11] Schwartz S Mumbach MR Jovanovic M Wang T Maciag K Bushkin GG. Perturbation of M6a writers reveals two distinct classes of mrna methylation at internal and 5' Sites. Cell Rep. (2014) 8:284–96. doi: 10.1016/j.celrep.2014.05.048 PMC4142486 24981863

[12] Huang E Chen L. Rna N(6)-methyladenosine modification in female reproductive biology and pathophysiology. Cell Commun Signal. (2023) 21:53. doi: 10.1186/s12964-023-01078-4 36894952 PMC9996912

[13] Barbieri I Tzelepis K Pandolfini L Shi J Millán-Zambrano G Robson SC. Promoter-bound mettl3 maintains myeloid leukaemia by M(6)a-dependent translation control. Nature. (2017) 552:126–31. doi: 10.1038/nature24678 PMC6217924 29186125

[14] Vu LP Pickering BF Cheng Y Zaccara S Nguyen D Minuesa G. The N(6)-methyladenosine (M(6)a)-forming enzyme mettl3 controls myeloid differentiation of normal hematopoietic and leukemia cells. Nat Med. (2017) 23:1369–76. doi: 10.1038/nm.4416 PMC5677536 28920958

[15] Chen M Wei L Law CT Tsang FH Shen J Cheng CL. Rna N6-methyladenosine methyltransferase-like 3 promotes liver cancer progression through ythdf2-dependent posttranscriptional silencing of socs2. Hepatology. (2018) 67:2254–70. doi: 10.1002/hep.29683 29171881

[16] He H Wu W Sun Z Chai L. Mir-4429 prevented gastric cancer progression through targeting mettl3 to inhibit M(6)a-caused stabilization of sec62. Biochem Biophys Res Commun. (2019) 517:581–7. doi: 10.1016/j.bbrc.2019.07.058 31395342

[17] Choe J Lin S Zhang W Liu Q Wang L Ramirez-Moya J. Mrna circularization by mettl3-eif3h enhances translation and promotes oncogenesis. Nature. (2018) 561:556–60. doi: 10.1038/s41586-018-0538-8 PMC6234840 30232453

[18] Yue B Song C Yang L Cui R Cheng X Zhang Z. Mettl3-mediated N6-methyladenosine modification is critical for epithelial-mesenchymal transition and metastasis of gastric cancer. Mol Cancer. (2019) 18:142. doi: 10.1186/s12943-019-1065-4 31607270 PMC6790244

[19] Han J Wang JZ Yang X Yu H Zhou R Lu HC. Mettl3 promote tumor proliferation of bladder cancer by accelerating pri-mir221/222 maturation in M6a-dependent manner. Mol Cancer. (2019) 18:110. doi: 10.1186/s12943-019-1036-9 31228940 PMC6588935

[20] Mu H Zhang T Yang Y Zhang D Gao J Li J. Mettl3-mediated mrna N(6)-methyladenosine is required for oocyte and follicle development in mice. Cell Death Dis. (2021) 12:989. doi: 10.1038/s41419-021-04272-9 34689175 PMC8542036

[21] Pan F Lin XR Hao LP Chu XY Wan HJ Wang R. The role of rna methyltransferase mettl3 in hepatocellular carcinoma: results and perspectives. Front Cell Dev Biol. (2021) 9:674919. doi: 10.3389/fcell.2021.674919 34046411 PMC8144501

[22] Wang T Kong S Tao M Ju S. The potential role of rna N6-methyladenosine in cancer progression. Mol Cancer. (2020) 19:88. doi: 10.1186/s12943-020-01204-7 32398132 PMC7216508

[23] Chen XY Zhang J Zhu JS. The role of M(6)a rna methylation in human cancer. Mol Cancer. (2019) 18:103. doi: 10.1186/s12943-019-1033-z 31142332 PMC6540575

[24] Sledz P Jinek M. Structural insights into the molecular mechanism of the M(6)a writer complex. Elife. (2016) 5:e18434. doi: 10.7554/eLife.18434 27627798 PMC5023411

[25] Wang P Doxtader KA Nam Y. Structural basis for cooperative function of mettl3 and mettl14 methyltransferases. Mol Cell. (2016) 63:306–17. doi: 10.1016/j.molcel.2016.05.041 PMC4958592 27373337

[26] Weng H Huang H Wu H Qin X Zhao BS Dong L. Mettl14 inhibits hematopoietic stem/progenitor differentiation and promotes leukemogenesis via mrna M(6)a modification. Cell Stem Cell. (2018) 22:191–205.e9. doi: 10.1016/j.stem.2017.11.016 29290617 PMC5860916

[27] Liu J Eckert MA Harada BT Liu SM Lu Z Yu K. M(6)a mrna methylation regulates akt activity to promote the proliferation and tumorigenicity of endometrial cancer. Nat Cell Biol. (2018) 20:1074–83. doi: 10.1038/s41556-018-0174-4 PMC6245953 30154548

[28] Ma JZ Yang F Zhou CC Liu F Yuan JH Wang F. Mettl14 suppresses the metastatic potential of hepatocellular carcinoma by modulating N(6) -methyladenosine-dependent primary microrna processing. Hepatology. (2017) 65:529–43. doi: 10.1002/hep.28885 27774652

[29] Panneerdoss S Eedunuri VK Yadav P Timilsina S Rajamanickam S Viswanadhapalli S. Cross-talk among writers, readers, and erasers of M(6)a regulates cancer growth and progression. Sci Adv. (2018) 4:eaar8263. doi: 10.1126/sciadv.aar8263 30306128 PMC6170038

[30] Jia GX Lin Z Yan RG Wang GW Zhang XN Li C. Wtap function in sertoli cells is essential for sustaining the spermatogonial stem cell niche. Stem Cell Rep. (2020) 15:968–82. doi: 10.1016/j.stemcr.2020.09.001 PMC7566211 33053361

[31] Bansal H Yihua Q Iyer SP Ganapathy S Proia DA Penalva LO. Wtap is a novel oncogenic protein in acute myeloid leukemia. Leukemia. (2014) 28:1171–4. doi: 10.1038/leu.2014.16 PMC4369791 24413322

[32] Wu LS Qian JY Wang M Yang H. Identifying the role of wilms tumor 1 associated protein in cancer prediction using integrative genomic analyses. Mol Med Rep. (2016) 14:2823–31. doi: 10.3892/mmr.2016.5528 27430156

[33] Barbolina MV Adley BP Shea LD Stack MS. Wilms tumor gene protein 1 is associated with ovarian cancer metastasis and modulates cell invasion. Cancer. (2008) 112:1632–41. doi: 10.1002/cncr.23341 18260155

[34] Li Q Wang C Dong W Su Y Ma Z. Wtap facilitates progression of endometrial cancer via cav-1/nf-Kb axis. Cell Biol Int. (2021) 45:1269–77. doi: 10.1002/cbin.11570 33559954

[35] Li Y Ge YZ Xu L Xu Z Dou Q Jia R. The potential roles of rna N6-methyladenosine in urological tumors. Front Cell Dev Biol. (2020) 8:579919. doi: 10.3389/fcell.2020.579919 33015074 PMC7510505

[36] Ruszkowska A. Mettl16, methyltransferase-like protein 16: current insights into structure and function. Int J Mol Sci. (2021) 22:2176. doi: 10.3390/ijms22042176 33671635 PMC7927073

[37] Satterwhite ER Mansfield KD. Rna methyltransferase mettl16: targets and function. Wiley Interdiscip Rev RNA. (2022) 13:e1681. doi: 10.1002/wrna.1681 34227247 PMC9286414

[38] Mendel M Chen KM Homolka D Gos P Pandey RR McCarthy AA. Methylation of structured rna by the M(6)a writer mettl16 is essential for mouse embryonic development. Mol Cell. (2018) 71:986–1000.e11. doi: 10.1016/j.molcel.2018.08.004 30197299 PMC6162343

[39] Zeng X Zhao F Cui G Zhang Y Deshpande RA Chen Y. Mettl16 antagonizes mre11-mediated DNA end resection and confers synthetic lethality to parp inhibition in pancreatic ductal adenocarcinoma. Nat Cancer. (2022) 3:1088–104. doi: 10.1038/s43018-022-00429-3 36138131

[40] Su R Dong L Li Y Gao M He PC Liu W. Mettl16 exerts an M(6)a-independent function to facilitate translation and tumorigenesis. Nat Cell Biol. (2022) 24:205–16. doi: 10.1038/s41556-021-00835-2 PMC9070413 35145225

[41] Wang XK Zhang YW Wang CM Li B Zhang TZ Zhou WJ. Mettl16 promotes cell proliferation by up-regulating cyclin D1 expression in gastric cancer. J Cell Mol Med. (2021) 25:6602–17. doi: 10.1111/jcmm.16664 PMC8278090 34075693

[42] Dai YZ Liu YD Li J Chen MT Huang M Wang F. Mettl16 promotes hepatocellular carcinoma progression through downregulating rab11b-as1 in an M(6)a-dependent manner. Cell Mol Biol Lett. (2022) 27:41. doi: 10.1186/s11658-022-00342-8 35596159 PMC9123709

[43] Han L Dong L Leung K Zhao Z Li Y Gao L. Mettl16 drives leukemogenesis and leukemia stem cell self-renewal by reprogramming bcaa metabolism. Cell Stem Cell. (2023) 30:52–68.e13. doi: 10.1016/j.stem.2022.12.006 36608679 PMC9838187

[44] Fedeles BI Singh V Delaney JC Li D Essigmann JM. The alkb family of fe(Ii)/alpha-ketoglutarate-dependent dioxygenases: repairing nucleic acid alkylation damage and beyond. J Biol Chem. (2015) 290:20734–42. doi: 10.1074/jbc.R115.656462 PMC4543635 26152727

[45] Yang Z Yu GL Zhu X Peng TH Lv YC. Critical roles of fto-mediated mrna M6a demethylation in regulating adipogenesis and lipid metabolism: implications in lipid metabolic disorders. Genes Dis. (2022) 9:51–61. doi: 10.1016/j.gendis.2021.01.005 35005107 PMC8720706

[46] Zarza-Rebollo JA Molina E Rivera M. The role of the fto gene in the relationship between depression and obesity. A systematic review. Neurosci Biobehav Rev. (2021) 127:630–7. doi: 10.1016/j.neubiorev.2021.05.013 34019853

[47] Jia G Fu Y Zhao X Dai Q Zheng G Yang Y. N6-methyladenosine in nuclear rna is a major substrate of the obesity-associated fto. Nat Chem Biol. (2011) 7:885–7. doi: 10.1038/nchembio.687 PMC3218240 22002720

[48] Linder B Grozhik AV Olarerin-George AO Meydan C Mason CE Jaffrey SR. Single-nucleotide-resolution mapping of M6a and M6am throughout the transcriptome. Nat Methods. (2015) 12:767–72. doi: 10.1038/nmeth.3453 PMC4487409 26121403

[49] Wei J Liu F Lu Z Fei Q Ai Y He PC. Differential M(6)a, M(6)a(M), and M(1)a demethylation mediated by fto in the cell nucleus and cytoplasm. Mol Cell. (2018) 71:973–85.e5. doi: 10.1016/j.molcel.2018.08.011 30197295 PMC6151148

[50] Zhang X Wei LH Wang Y Xiao Y Liu J Zhang W. Structural insights into fto's catalytic mechanism for the demethylation of multiple rna substrates. Proc Natl Acad Sci U S A. (2019) 116:2919–24. doi: 10.1073/pnas.1820574116 PMC6386707 30718435

[51] Zou D Dong L Li C Yin Z Rao S Zhou Q. The M(6)a eraser fto facilitates proliferation and migration of human cervical cancer cells. Cancer Cell Int. (2019) 19:321. doi: 10.1186/s12935-019-1045-1 31827395 PMC6888952

[52] Wang T Li W Ye B Zhang S Lei X Zhang D. Fto-stabilized lncrna hoxc13-as epigenetically upregulated fzd6 and activated wnt/B-catenin signaling to drive cervical cancer proliferation, invasion, and emt. J buon. (2021) 26:1279–91. 34564982

[53] Qu J Hou Y Chen Q Chen J Li Y Zhang E. Rna demethylase alkbh5 promotes tumorigenesis in multiple myeloma via traf1-mediated activation of nf-kappab and mapk signaling pathways. Oncogene. (2022) 41:400–13. doi: 10.1038/s41388-021-02095-8 PMC8755544 34759347

[54] He Y Yue H Cheng Y Ding Z Xu Z Lv C. Alkbh5-mediated M(6)a demethylation of kcnk15-as1 inhibits pancreatic cancer progression via regulating kcnk15 and pten/akt signaling. Cell Death Dis. (2021) 12:1121. doi: 10.1038/s41419-021-04401-4 34853296 PMC8636648

[55] Jiang Y Wan Y Gong M Zhou S Qiu J Cheng W. Rna demethylase alkbh5 promotes ovarian carcinogenesis in a simulated tumour microenvironment through stimulating nf-Kb pathway. J Cell Mol Med. (2020) 24:6137–48. doi: 10.1111/jcmm.15228 PMC7294121 32329191

[56] Zhang C Samanta D Lu H Bullen JW Zhang H Chen I. Hypoxia induces the breast cancer stem cell phenotype by hif-dependent and alkbh5-mediated M^6^a-demethylation of nanog mrna. Proc Natl Acad Sci U S A. (2016) 113:E2047–56. doi: 10.1073/pnas.1602883113 PMC4833258 27001847

[57] Zhang S Zhao BS Zhou A Lin K Zheng S Lu Z. M(6)a demethylase alkbh5 maintains tumorigenicity of glioblastoma stem-like cells by sustaining foxm1 expression and cell proliferation program. Cancer Cell. (2017) 31:591–606.e6. doi: 10.1016/j.ccell.2017.02.013 28344040 PMC5427719

[58] Alarcon CR Goodarzi H Lee H Liu X Tavazoie S Tavazoie SF. Hnrnpa2b1 is a mediator of M(6)a-dependent nuclear rna processing events. Cell. (2015) 162:1299–308. doi: 10.1016/j.cell.2015.08.011 PMC4673968 26321680

[59] Huang H Weng H Sun W Qin X Shi H Wu H. Recognition of rna N(6)-methyladenosine by igf2bp proteins enhances mrna stability and translation. Nat Cell Biol. (2018) 20:285–95. doi: 10.1038/s41556-018-0045-z PMC5826585 29476152

[60] Chen Z Zhong X Xia M Zhong J. The roles and mechanisms of the M6a reader protein ythdf1 in tumor biology and human diseases. Mol Ther Nucleic Acids. (2021) 26:1270–9. doi: 10.1016/j.omtn.2021.10.023 PMC8609105 34853726

[61] Shi H Wang X Lu Z Zhao BS Ma H Hsu PJ. Ythdf3 facilitates translation and decay of N(6)-methyladenosine-modified rna. Cell Res. (2017) 27:315–28. doi: 10.1038/cr.2017.15 PMC5339834 28106072

[62] Li A Chen YS Ping XL Yang X Xiao W Yang Y. Cytoplasmic M(6)a reader ythdf3 promotes mrna translation. Cell Res. (2017) 27:444–7. doi: 10.1038/cr.2017.10 PMC5339832 28106076

[63] Li J Wu L Pei M Zhang Y. Ythdf2, a protein repressed by mir-145, regulates proliferation, apoptosis, and migration in ovarian cancer cells. J Ovarian Res. (2020) 13:111. doi: 10.1186/s13048-020-00717-5 32948220 PMC7501604

[64] Li J Meng S Xu M Wang S He L Xu X. Downregulation of N(6)-methyladenosine binding ythdf2 protein mediated by mir-493-3p suppresses prostate cancer by elevating N(6)-methyladenosine levels. Oncotarget. (2018) 9:3752–64. doi: 10.18632/oncotarget.23365 PMC5790497 29423080

[65] Li Y Lu T Wang J Zhuo Z Miao L Yang Z. Ythdc1 gene polymorphisms and neuroblastoma susceptibility in chinese children. Aging (Albany NY). (2021) 13:25426–39. doi: 10.18632/aging.203760 PMC8714171 34897032

[66] Roundtree IA Luo GZ Zhang Z Wang X Zhou T Cui Y. Ythdc1 mediates nuclear export of N(6)-Methyladenosine methylated mrnas. Elife. (2017), 6. doi: 10.7554/eLife.31311 PMC5648532 28984244

[67] Widagdo J Anggono V Wong JJ. The multifaceted effects of ythdc1-mediated nuclear M(6)a recognition. Trends Genet. (2022) 38:325–32. doi: 10.1016/j.tig.2021.11.005 34920906

[68] Liu R Kasowitz SD Homolka D Leu NA Shaked JT Ruthel G. Ythdc2 is essential for pachytene progression and prevents aberrant microtubule-driven telomere clustering in male meiosis. Cell Rep. (2021) 37:110110. doi: 10.1016/j.celrep.2021.110110 34910909 PMC8720241

[69] Hsu PJ Zhu Y Ma H Guo Y Shi X Liu Y. Ythdc2 is an N(6)-methyladenosine binding protein that regulates mammalian spermatogenesis. Cell Res. (2017) 27:1115–27. doi: 10.1038/cr.2017.99 PMC5587856 28809393

[70] Zhou KI Shi H Lyu R Wylder AC Matuszek Z Pan JN. Regulation of co-transcriptional pre-mrna splicing by M(6)a through the low-complexity protein hnrnpg. Mol Cell. (2019) 76:70–81 e9. doi: 10.1016/j.molcel.2019.07.005 31445886 PMC6778029

[71] Jiang F Tang X Tang C Hua Z Ke M Wang C. Hnrnpa2b1 promotes multiple myeloma progression by increasing akt3 expression via M6a-dependent stabilization of ilf3 mrna. J Hematol Oncol. (2021) 14:54. doi: 10.1186/s13045-021-01066-6 33794982 PMC8017865

[72] Korn SM Ulshofer CJ Schneider T Schlundt A. Structures and target rna preferences of the rna-binding protein family of igf2bps: an overview. Structure. (2021) 29:787–803. doi: 10.1016/j.str.2021.05.001 34022128

[73] Nielsen J Lykke-Andersen J Wewer UM. A family of insulin-like growth factor ii mrna-binding proteins represses translation in late development. Mol Cell Biol. (1999) 19:1262–70. doi: 10.1128/MCB.19.2.1262 PMC116055 9891060

[74] Degrauwe N Suvà ML Janiszewska M Riggi N Stamenkovic I. Imps: an rna-binding protein family that provides a link between stem cell maintenance in normal development and cancer. Genes Dev. (2016) 30:2459–74. doi: 10.1101/gad.287540.116 PMC5159662 27940961

[75] Edens BM Vissers C Su J Arumugam S Xu Z Shi H. Fmrp modulates neural differentiation through M(6)a-dependent mrna nuclear export. Cell Rep. (2019) 28:845–54 e5. doi: 10.1016/j.celrep.2019.06.072 31340148 PMC6687293

[76] Hsu PJ Shi H Zhu AC Lu Z Miller N Edens BM. The rna-binding protein fmrp facilitates the nuclear export of N(6)-methyladenosine-containing mrnas. J Biol Chem. (2019) 294:19889–95. doi: 10.1074/jbc.AC119.010078 PMC6937581 31753916

[77] Bourgeois CF Mortreux F Auboeuf D. The multiple functions of rna helicases as drivers and regulators of gene expression. Nat Rev Mol Cell Biol. (2016) 17:426–38. doi: 10.1038/nrm.2016.50 27251421

[78] Ianniello Z Sorci M Ceci Ginistrelli L Iaiza A Marchioni M Tito C. New insight into the catalytic -dependent and -independent roles of mettl3 in sustaining aberrant translation in chronic myeloid leukemia. Cell Death Dis. (2021) 12:870. doi: 10.1038/s41419-021-04169-7 34561421 PMC8463696

[79] Mao Y Dong L Liu XM Guo J Ma H Shen B. M(6)a in mrna coding regions promotes translation via the rna helicase-containing ythdc2. Nat Commun. (2019) 10:5332. doi: 10.1038/s41467-019-13317-9 31767846 PMC6877647

[80] Chang G Shi L Ye Y Shi H Zeng L Tiwary S. Ythdf3 induces the translation of M(6)a-enriched gene transcripts to promote breast cancer brain metastasis. Cancer Cell. (2020) 38:857–71 e7. doi: 10.1016/j.ccell.2020.10.004 33125861 PMC7738369

[81] Liu T Wei Q Jin J Luo Q Liu Y Yang Y. The M6a reader ythdf1 promotes ovarian cancer progression via augmenting eif3c translation. Nucleic Acids Res. (2020) 48:3816–31. doi: 10.1093/nar/gkaa048 PMC7144925 31996915

[82] Orouji E Peitsch WK Orouji A Houben R Utikal J. Oncogenic role of an epigenetic reader of M(6)a rna modification: ythdf1 in merkel cell carcinoma. Cancers (Basel). (2020) 12:202. doi: 10.3390/cancers12010202 31947544 PMC7016651

[83] Kretschmer J Hackert P Sloan KE Bohnsack MT. The M(6)a reader protein ythdc2 interacts with the small ribosomal subunit and the 5 ‘-3 ’ Exoribonuclease xrn1. RNA. (2018) 24:1339–50. doi: 10.1261/rna.064238.117 PMC6140455 29970596

[84] Wojtas MN Pandey RR Mendel M Homolka D Sachidanandam R Pillai RS. Regulation of M(6)a transcripts by the 3'–>5' Rna helicase ythdc2 is essential for a successful meiotic program in the mammalian germline. Mol Cell. (2017) 68:374–87 e12. doi: 10.1016/j.molcel.2017.09.021 29033321

[85] Geula S Moshitch-Moshkovitz S Mansour AA Kol N Hershkovitz V Peer E. M6a mrna methylation facilitates resolution of naive pluripotency toward differentiation. Science. (2015) 347:1002–6. doi: 10.1126/science.1261417 25569111

[86] Edupuganti RR Geiger S Lindeboom RGH Shi H Hsu PJ Lu Z. N(6)-methyladenosine (M(6)a) recruits and repels proteins to regulate mrna homeostasis. Nat Struct Mol Biol. (2017) 24:870–8. doi: 10.1038/nsmb.3462 PMC5725193 28869609

[87] Zhang F Kang Y Wang M Li Y Xu T Yang W. Fragile X mental retardation protein modulates the stability of its M6a-marked messenger rna targets. Hum Mol Genet. (2018) 27:3936–50. doi: 10.1093/hmg/ddy292 PMC6216232 30107516

[88] Huang H Weng H Sun W Qin X Shi H Wu H. Publisher correction: recognition of rna N(6)-methyladenosine by igf2bp proteins enhances mrna stability and translation. Nat Cell Biol. (2020) 22:1288. doi: 10.1038/s41556-020-00580-y 32855523

[89] Luo Y Na Z Slavoff SA. P-bodies: Composition, Properties, and Functions. Biochemistry. (2018) 57:2424–31. doi: 10.1021/acs.biochem.7b01162 PMC6296482 29381060

[90] Zheng D Ezzeddine N Chen CY Zhu W He X Shyu AB. Deadenylation is prerequisite for P-body formation and mrna decay in mammalian cells. J Cell Biol. (2008) 182:89–101. doi: 10.1083/jcb.200801196 18625844 PMC2447901

[91] Su R Dong L Li C Nachtergaele S Wunderlich M Qing Y. R-2hg exhibits anti-tumor activity by targeting fto/M(6)a/myc/cebpa signaling. Cell. (2018) 172:90–105 e23. doi: 10.1016/j.cell.2017.11.031 29249359 PMC5766423

[92] Moor RM Dai Y Lee C Fulka J Jr. Oocyte maturation and embryonic failure. Hum Reprod Update. (1998) 4:223–36. doi: 10.1093/humupd/4.3.223 9741707

[93] Gindi N Grossman H Bar-Joseph H Miller I Nemerovsky L Hadas R. Fyn and argonaute 2 participate in maternal-mrna degradation during mouse oocyte maturation. Cell Cycle. (2022) 21:792–804. doi: 10.1080/15384101.2022.2031427 35104175 PMC8973342

[94] Qi ST Ma JY Wang ZB Guo L Hou Y Sun QY. N6-methyladenosine sequencing highlights the involvement of mrna methylation in oocyte meiotic maturation and embryo development by regulating translation in xenopus laevis. J Biol Chem. (2016) 291:23020–6. doi: 10.1074/jbc.M116.748889 PMC5087722 27613873

[95] Ivanova I Much C Di Giacomo M Azzi C Morgan M Moreira PN. The rna M(6)a reader ythdf2 is essential for the post-transcriptional regulation of the maternal transcriptome and oocyte competence. Mol Cell. (2017) 67:1059–67.e4. doi: 10.1016/j.molcel.2017.08.003 28867294 PMC5613143

[96] Xia H Zhong C Wu X Chen J Tao B Xia X. Mettl3 mutation disrupts gamete maturation and reduces fertility in zebrafish. Genetics. (2018) 208:729–43. doi: 10.1534/genetics.117.300574 PMC5788534 29196300

[97] Wu Y Xu X Qi M Chen C Li M Yan R. N(6)-methyladenosine regulates maternal rna maintenance in oocytes and timely rna decay during mouse maternal-to-zygotic transition. Nat Cell Biol. (2022) 24:917–27. doi: 10.1038/s41556-022-00915-x 35606490

[98] Hao J Hu H Jiang Z Yu X Li C Chen L. Microrna-670 modulates igf2bp1 expression to regulate rna methylation in parthenogenetic mouse embryonic development. Sci Rep. (2020) 10:4782. doi: 10.1038/s41598-020-61816-3 32179813 PMC7076016

[99] Cao Z Zhang D Wang Y Tong X Avalos LFC Khan IM. Identification and functional annotation of M6a methylation modification in granulosa cells during antral follicle development in pigs. Anim Reprod Sci. (2020) 219:106510. doi: 10.1016/j.anireprosci.2020.106510 32828396

[100] Li L Zheng P Dean J. Maternal control of early mouse development. Development. (2010) 137:859–70. doi: 10.1242/dev.039487 PMC2834456 20179092

[101] Lubzens E Young G Bobe J Cerda J. Oogenesis in teleosts: how eggs are formed. Gen Comp Endocrinol. (2010) 165:367–89. doi: 10.1016/j.ygcen.2009.05.022 19505465

[102] Yu XX Liu YH Liu XM Wang PC Liu S Miao JK. Ascorbic acid induces global epigenetic reprogramming to promote meiotic maturation and developmental competence of porcine oocytes. Sci Rep. (2018) 8:6132. doi: 10.1038/s41598-018-24395-y 29666467 PMC5904140

[103] Kasowitz SD Ma J Anderson SJ Leu NA Xu Y Gregory BD. Nuclear M6a reader ythdc1 regulates alternative polyadenylation and splicing during mouse oocyte development. PloS Genet. (2018) 14:e1007412. doi: 10.1371/journal.pgen.1007412 29799838 PMC5991768

[104] Bailey AS Batista PJ Gold RS Chen YG de Rooij DG Chang HY. The conserved rna helicase ythdc2 regulates the transition from proliferation to differentiation in the germline. Elife. (2017) 6:e26116. doi: 10.7554/eLife.26116 29087293 PMC5703642

[105] Zeng M Dai X Liang Z Sun R Huang S Luo L. Critical roles of mrna M(6)a modification and ythdc2 expression for meiotic initiation and progression in female germ cells. Gene. (2020) 753:144810. doi: 10.1016/j.gene.2020.144810 32470506

[106] Zhao X Tian GG Fang Q Pei X Wang Z Wu J. Comparison of rna M(6)a and DNA methylation profiles between mouse female germline stem cells and sto cells. Mol Ther Nucleic Acids. (2021) 23:431–9. doi: 10.1016/j.omtn.2020.11.020 PMC7803632 33473328

[107] Kontur C Jeong M Cifuentes D Giraldez AJ. Ythdf M(6)a readers function redundantly during zebrafish development. Cell Rep. (2020) 33:108598. doi: 10.1016/j.celrep.2020.108598 33378672 PMC11407899

[108] Ma Z Li Q Liu P Dong W Zuo Y. Mettl3 regulates M6a in endometrioid epithelial ovarian cancer independently of mettl14 and wtap. Cell Biol Int. (2020) 44:2524–31. doi: 10.1002/cbin.11459 32869897

[109] Hao L Wang JM Liu BQ Yan J Li C Jiang JY. M6a-ythdf1-mediated trim29 upregulation facilitates the stem cell-like phenotype of cisplatin-resistant ovarian cancer cells. Biochim Biophys Acta Mol Cell Res. (2021) 1868:118878. doi: 10.1016/j.bbamcr.2020.118878 33011193

[110] Pendleton KE Chen B Liu K Hunter OV Xie Y Tu BP. The U6 snrna M(6)a methyltransferase mettl16 regulates sam synthetase intron retention. Cell. (2017) 169:824–35 e14. doi: 10.1016/j.cell.2017.05.003 28525753 PMC5502809

[111] Zou S Toh JD Wong KH Gao YG Hong W Woon EC. N(6)-methyladenosine: A conformational marker that regulates the substrate specificity of human demethylases fto and alkbh5. Sci Rep. (2016) 6:25677. doi: 10.1038/srep25677 27156733 PMC4860565

[112] Galganski L Urbanek MO Krzyzosiak WJ. Nuclear speckles: molecular organization, biological function and role in disease. Nucleic Acids Res. (2017) 45:10350–68. doi: 10.1093/nar/gkx759 PMC5737799 28977640

[113] Zhao X Yang Y Sun BF Shi Y Yang X Xiao W. Fto-dependent demethylation of N6-methyladenosine regulates mrna splicing and is required for adipogenesis. Cell Res. (2014) 24:1403–19. doi: 10.1038/cr.2014.151 PMC4260349 25412662

[114] Liu HB Muhammad T Guo Y Li MJ Sha QQ Zhang CX. Rna-binding protein igf2bp2/imp2 is a critical maternal activator in early zygotic genome activation. Adv Sci (Weinh). (2019) 6:1900295. doi: 10.1002/advs.201900295 31406667 PMC6685478

[115] Ren F Lin Q Gong G Du X Dan H Qin W. Igf2bp3 maintains maternal rna stability and ensures early embryo development in zebrafish. Commun Biol. (2020) 3:94. doi: 10.1038/s42003-020-0827-2 32127635 PMC7054421

[116] Barton SJ Mosquera M Cleal JK Fuller AS Crozier SR Cooper C. Relation of fto gene variants to fetal growth trajectories: findings from the southampton women's survey. Placenta. (2016) 38:100–6. doi: 10.1016/j.placenta.2015.12.015 PMC4776702 26907388

[117] Taniguchi K Kawai T Kitawaki J Tomikawa J Nakabayashi K Okamura K. Epitranscriptomic profiling in human placenta: N6-methyladenosine modification at the 5'-untranslated region is related to fetal growth and preeclampsia. FASEB J. (2020) 34:494–512. doi: 10.1096/fj.201900619RR 31914637 PMC7027905

[118] Bassols J Prats-Puig A Vazquez-Ruiz M Garcia-Gonzalez MM Martinez-Pascual M Avelli P. Placental fto expression relates to fetal growth. Int J Obes (Lond). (2010) 34:1365–70. doi: 10.1038/ijo.2010.62 20351740

[119] Song T Lu J Deng Z Xu T Yang Y Wei H. Maternal obesity aggravates the abnormality of porcine placenta by increasing N(6)-methyladenosine. Int J Obes (Lond). (2018) 42:1812–20. doi: 10.1038/s41366-018-0113-2 29795472

[120] Mor G Aldo P Alvero AB. The unique immunological and microbial aspects of pregnancy. Nat Rev Immunol. (2017) 17:469–82. doi: 10.1038/nri.2017.64 28627518

[121] Tang L Wei X Li T Chen Y Dai Z Lu C. Emerging perspectives of rna N(6)-methyladenosine (M(6)a) modification on immunity and autoimmune diseases. Front Immunol. (2021) 12:630358. doi: 10.3389/fimmu.2021.630358 33746967 PMC7973041

[122] Wang H Hu X Huang M Liu J Gu Y Ma L. Mettl3-mediated mrna M(6)a methylation promotes dendritic cell activation. Nat Commun. (2019) 10:1898. doi: 10.1038/s41467-019-09903-6 31015515 PMC6478715

[123] Tong J Wang X Liu Y Ren X Wang A Chen Z. Pooled crispr screening identifies M(6)a as a positive regulator of macrophage activation. Sci Adv. (2021) 7:eabd4742. doi: 10.1126/sciadv.abd4742 33910903 PMC8081357

[124] Dong L Chen C Zhang Y Guo P Wang Z Li J. The loss of rna N(6)-adenosine methyltransferase mettl14 in tumor-associated macrophages promotes cd8(+) T cell dysfunction and tumor growth. Cancer Cell. (2021) 39:945–57 e10. doi: 10.1016/j.ccell.2021.04.016 34019807

[125] Ma S Yan J Barr T Zhang J Chen Z Wang LS. The rna M6a reader ythdf2 controls nk cell antitumor and antiviral immunity. J Exp Med. (2021) 218:e20210279. doi: 10.1084/jem.20210279 34160549 PMC8225680

[126] Huang H Weng H Chen J. M(6)a modification in coding and non-coding rnas: roles and therapeutic implications in cancer. Cancer Cell. (2020) 37:270–88. doi: 10.1016/j.ccell.2020.02.004 PMC7141420 32183948

[127] Deng X Su R Weng H Huang H Li Z Chen J. Rna N(6)-methyladenosine modification in cancers: current status and perspectives. Cell Res. (2018) 28:507–17. doi: 10.1038/s41422-018-0034-6 PMC5951805 29686311

[128] Dong S Wu Y Liu Y Weng H Huang H. N(6) -methyladenosine steers rna metabolism and regulation in cancer. Cancer Commun (Lond). (2021) 41:538–59. doi: 10.1002/cac2.12161 PMC8286143 33955720

[129] Yang Y Hsu PJ Chen YS Yang YG. Dynamic transcriptomic M(6)a decoration: writers, erasers, readers and functions in rna metabolism. Cell Res. (2018) 28:616–24. doi: 10.1038/s41422-018-0040-8 PMC5993786 29789545

[130] Shi H Wei J He C. Where, when, and how: context-dependent functions of rna methylation writers, readers, and erasers. Mol Cell. (2019) 74:640–50. doi: 10.1016/j.molcel.2019.04.025 PMC6527355 31100245

[131] Li Q Li X Tang H Jiang B Dou Y Gorospe M. Nsun2-mediated M5c methylation and mettl3/mettl14-mediated M6a methylation cooperatively enhance P21 translation. J Cell Biochem. (2017) 118:2587–98. doi: 10.1002/jcb.25957 PMC5509477 28247949

[132] Ding C Zou Q Ding J Ling M Wang W Li H. Increased N6-methyladenosine causes infertility is associated with fto expression. J Cell Physiol. (2018) 233:7055–66. doi: 10.1002/jcp.26507 PMC13482491 29384212

[133] Tang Z Ju Y Dai X Ni N Liu Y Zhang D. Ho-1-mediated ferroptosis as a target for protection against retinal pigment epithelium degeneration. Redox Biol. (2021) 43:101971. doi: 10.1016/j.redox.2021.101971 33895485 PMC8099560

[134] Gu Y Chu X Morgan JA Lewis DF Wang Y. Upregulation of mettl3 expression and M6a rna methylation in placental trophoblasts in preeclampsia. Placenta. (2021) 103:43–9. doi: 10.1016/j.placenta.2020.10.016 33070036

[135] Li R Qiu X He M Qiao J He J Zhong M. Retracted article: mettl3-mediated mature mir-497-5p/195-5p inhibits trophoblast migration and invasion by targeting wwp1 in preeclampsia. Cell Cycle. (2022) 21:iii–xviii. doi: 10.1080/15384101.2021.1982527 34592887 PMC9345615

[136] Zhang Y Yang H Long Y Zhang Y Chen R Shi J. Circrna N6-methyladenosine methylation in preeclampsia and the potential role of N6-methyladenosine-modified circpappa2 in trophoblast invasion. Sci Rep. (2021) 11:24357. doi: 10.1038/s41598-021-03662-5 34934095 PMC8692596

[137] Fan W Zhou W Yan Q Peng Y Wang H Kong C. Upregulation of mettl14 contributes to trophoblast dysfunction by elevating foxo3a expression in an M(6)a-dependent manner. Placenta. (2022) 124:18–27. doi: 10.1016/j.placenta.2022.05.008 35597169

[138] Guo Y Song W Yang Y. Inhibition of alkbh5-mediated M(6) a modification of pparg mrna alleviates H/R-induced oxidative stress and apoptosis in placenta trophoblast. Environ Toxicol. (2022) 37:910–24. doi: 10.1002/tox.23454 34995009

[139] Yang Q Ma Y Liu Y Shao X Jia W Yu X. Mnsfbeta regulates placental development by conjugating igf2bp2 to enhance trophoblast cell invasiveness. Cell Prolif. (2021) 54:e13145. doi: 10.1111/cpr.13145 34668606 PMC8666274

[140] Wang D Guan H Xia Y. Ythdc1 maintains trophoblasts function by promoting degradation of M6a-modified circmpp1. Biochem Pharmacol. (2023) 210:115456. doi: 10.1016/j.bcp.2023.115456 36780989

[141] Zheng Q Gan H Yang F Yao Y Hao F Hong L. Cytoplasmic M(1)a reader ythdf3 inhibits trophoblast invasion by downregulation of M(1)a-methylated igf1r. Cell Discov. (2020) 6:12. doi: 10.1038/s41421-020-0144-4 32194978 PMC7062805

[142] Qin X Chen Y Chen S Liu X Zeng W Tian F. Plasmacytoma variant translocation 1 (Pvt1) regulates trophoblast viability, proliferation, and migration and is downregulated in spontaneous abortion. Am J Reprod Immunol. (2019) 81:e13048. doi: 10.1111/aji.13048 30295989

[143] Huppertz B. Traditional and new routes of trophoblast invasion and their implications for pregnancy diseases. Int J Mol Sci. (2019) 21:289. doi: 10.3390/ijms21010289 31906245 PMC6981830

[144] Li XC Jin F Wang BY Yin XJ Hong W Tian FJ. The M6a demethylase alkbh5 controls trophoblast invasion at the maternal-fetal interface by regulating the stability of cyr61 mrna. Theranostics. (2019) 9:3853–65. doi: 10.7150/thno.31868 PMC6587351 31281518

[145] Zhang J Qiu Q Wang H Chen C Luo D. Trim46 contributes to high glucose-induced ferroptosis and cell growth inhibition in human retinal capillary endothelial cells by facilitating gpx4 ubiquitination. Exp Cell Res. (2021) 407:112800. doi: 10.1016/j.yexcr.2021.112800 34487731

[146] She J Tan K Liu J Cao S Li Z Peng Y. The alteration of M(6)a modification at the transcriptome-wide level in human villi during spontaneous abortion in the first trimester. Front Genet. (2022) 13:861853. doi: 10.3389/fgene.2022.861853 35754822 PMC9215105

[147] Chapron C Marcellin L Borghese B Santulli P. Rethinking mechanisms, diagnosis and management of endometriosis. Nat Rev Endocrinol. (2019) 15:666–82. doi: 10.1038/s41574-019-0245-z 31488888

[148] Giudice LC Kao LC. Endometriosis. Lancet. (2004) 364:1789–99. doi: 10.1016/S0140-6736(04)17403-5 15541453

[149] Vercellini P Vigano P Somigliana E Fedele L. Endometriosis: pathogenesis and treatment. Nat Rev Endocrinol. (2014) 10:261–75. doi: 10.1038/nrendo.2013.255 24366116

[150] Jiang L Wu J Wang S Yang X Yi M Zhang X. Exploring diagnostic M6a regulators in endometriosis. Aging (Albany NY). (2020) 12:25916–38. doi: 10.18632/aging.202163 PMC7803542 33232273

[151] Li X Xiong W Long X Dai X Peng Y Xu Y. Inhibition of mettl3/M6a/mir126 promotes the migration and invasion of endometrial stromal cells in endometriosisdagger. Biol Reprod. (2021) 105:1221–33. doi: 10.1093/biolre/ioab152 PMC10308507 34382070

[152] Szubert M Koziróg E Olszak O Krygier-Kurz K Kazmierczak J Wilczynski J. Adenomyosis and infertility—Review of medical and surgical approaches. Int J Environ Res Public Health. (2021) 18:1235. doi: 10.3390/ijerph18031235 33573117 PMC7908401

[153] Bulun SE Yildiz S Adli M Wei J-J. Adenomyosis pathogenesis: insights from next-generation sequencing. Hum Reprod Update. (2021) 27:1086–97. doi: 10.1093/humupd/dmab017 PMC8543024 34131719

[154] Cunningham RK Horrow MM Smith RJ Springer J. Adenomyosis: A sonographic diagnosis. RadioGraphics. (2018) 38:1576–89. doi: 10.1148/rg.2018180080 30207945

[155] Zhai J Li S Sen S Opoku-Anane J Du Y Chen ZJ. M(6)a rna methylation regulators contribute to eutopic endometrium and myometrium dysfunction in adenomyosis. Front Genet. (2020) 11:716. doi: 10.3389/fgene.2020.00716 32719721 PMC7350935

[156] Wang Q Wei Y Zhang J. Combined knockdown of D-dopachrome tautomerase and migration inhibitory factor inhibits the proliferation, migration, and invasion in human cervical cancer. Int J Gynecol Cancer. (2017) 27:634–42. doi: 10.1097/IGC.0000000000000951 28338494

[157] Jeanes YM Reeves S. Metabolic consequences of obesity and insulin resistance in polycystic ovary syndrome: diagnostic and methodological challenges. Nutr Res Rev. (2017) 30:97–105. doi: 10.1017/S0954422416000287 28222828

[158] Escobar-Morreale HF. Polycystic ovary syndrome: definition, aetiology, diagnosis and treatment. Nat Rev Endocrinol. (2018) 14:270–84. doi: 10.1038/nrendo.2018.24 29569621

[159] Schulte MM Tsai JH Moley KH. Obesity and pcos: the effect of metabolic derangements on endometrial receptivity at the time of implantation. Reprod Sci. (2015) 22:6–14. doi: 10.1177/1933719114561552 25488942 PMC4275454

[160] Snider AP Wood JR. Obesity induces ovarian inflammation and reduces oocyte quality. Reproduction. (2019) 158:R79–R90. doi: 10.1530/rep 30999278

[161] Lin Z Niu Y Wan A Chen D Liang H Chen X. Rna M(6) a methylation regulates sorafenib resistance in liver cancer through foxo3-mediated autophagy. EMBO J. (2020) 39:e103181. doi: 10.15252/embj.2019103181 32368828 PMC7298296

[162] Zhou L Han X Li W Wang N Yao L Zhao Y. N6-methyladenosine demethylase fto induces the dysfunctions of ovarian granulosa cells by upregulating flotillin 2. Reprod Sci. (2022) 29:1305–15. doi: 10.1007/s43032-021-00664-6 34254281

[163] Morgan S Anderson RA Gourley C Wallace WH Spears N. How do chemotherapeutic agents damage the ovary? Hum Reprod Update. (2012) 18:525–35. doi: 10.1093/humupd/dms022 22647504

[164] Brodie MJ Mintzer S Pack AM Gidal BE Vecht CJ Schmidt D. Enzyme induction with antiepileptic drugs: cause for concern? Epilepsia. (2013) 54:11–27. doi: 10.1111/j.1528-1167.2012.03671.x 23016553

[165] Blumenfeld Z. Preservation of fertility and ovarian function and minimalization of chemotherapy associated gonadotoxicity and premature. Mol Cell Endocrinol. (2002) 187:93–105. doi: 10.1016/S0303-7207(01)00712-2 11988316

[166] Huang B Ding C Zou Q Wang W Li H. Cyclophosphamide regulates N6-methyladenosine and M6a rna enzyme levels in human granulosa cells and in ovaries of a premature ovarian aging mouse model. Front Endocrinol (Lausanne). (2019) 10:415. doi: 10.3389/fendo.2019.00415 31316467 PMC6610338

[167] Zheng G Dahl JA Niu Y Fedorcsak P Huang CM Li CJ. Alkbh5 is a mammalian rna demethylase that impacts rna metabolism and mouse fertility. Mol Cell. (2013) 49:18–29. doi: 10.1016/j.molcel.2012.10.015 23177736 PMC3646334

[168] Zhao J Lu L. Interplay between rna methylation eraser fto and writer mettl3 in renal clear cell carcinoma patient survival. Recent Pat Anticancer Drug Discov. (2021) 16:363–76. doi: 10.2174/1574892816666210204125155 33563180

[169] Colombo N Creutzberg C Amant F Bosse T Gonzalez-Martin A Ledermann J. Esmo-esgo-estro consensus conference on endometrial cancer: diagnosis, treatment and follow-up. Ann Oncol. (2016) 27:16–41. doi: 10.1093/annonc/mdv484 26634381

[170] Kong A Johnson N Kitchener HC Lawrie TA. Adjuvant radiotherapy for stage I endometrial cancer: an updated cochrane systematic review and meta-analysis. J Natl Cancer Inst. (2012) 104:1625–34. doi: 10.1093/jnci/djs374 22962693

[171] McMeekin DS. Where is the future of endometrial cancer therapy? Ann Oncol. (2009) 20:1757–61. doi: 10.1093/annonc/mdp493 19861581

[172] Montejo M Werner TL Gaffney D. Current challenges in clinical management of endometrial cancer. Adv Drug Delivery Rev. (2009) 61:883–9. doi: 10.1016/j.addr.2009.04.014 19422864

[173] Gu J Bi F. Significance of N6-methyladenosine rna methylation regulators in immune infiltrates of ovarian cancer. Front Genet. (2021) 12:671179. doi: 10.3389/fgene.2021.671179 34306015 PMC8295008

[174] Zhang L Wan Y Zhang Z Jiang Y Gu Z Ma X. Igf2bp1 overexpression stabilizes peg10 mrna in an M6a-dependent manner and promotes endometrial cancer progression. Theranostics. (2021) 11:1100–14. doi: 10.7150/thno.49345 PMC7738899 33391523

[175] Nie X Tan J. N6-methyladenosine-related lncrnas is a potential marker for predicting prognosis and immunotherapy in ovarian cancer. Hereditas. (2022) 159:17. doi: 10.1186/s41065-022-00222-3 35303965 PMC8933961

[176] Xue T Liu X Zhang M Liu S Zou M Li Y. Padi2-catalyzed mek1 citrullination activates erk1/2 and promotes igf2bp1-mediated sox2 mrna stability in endometrial cancer. Adv Sci (Weinh). (2021) 8:2002831. doi: 10.1002/advs.202002831 33747724 PMC7967072

[177] Bhan A Soleimani M Mandal SS. Long noncoding rna and cancer: A new paradigm. Cancer Res. (2017) 77:3965–81. doi: 10.1158/0008-5472.CAN-16-2634 PMC8330958 28701486

[178] Zhang C Liu J Guo H Hong D Ji J Zhang Q. M6a rna methylation regulators were associated with the Malignancy and prognosis of ovarian cancer. Bioengineered. (2021) 12:3159–76. doi: 10.1080/21655979.2021.1946305 PMC8806923 34187307

[179] Chen G Liu B Yin S Li S Guo Y Wang M. Hypoxia induces an endometrial cancer stem-like cell phenotype via hif-dependent demethylation of sox2 mrna. Oncogenesis. (2020) 9:81. doi: 10.1038/s41389-020-00265-z 32913192 PMC7484801

[180] Li Q Ren CC Chen YN Yang L Zhang F Wang BJ. A risk score model incorporating three M6a rna methylation regulators and a related network of mirnas-M6a regulators-M6a target genes to predict the prognosis of patients with ovarian cancer. Front Cell Dev Biol. (2021) 9:703969. doi: 10.3389/fcell.2021.703969 34631700 PMC8495156

[181] Pu X Gu Z Gu Z. Alkbh5 regulates igf1r expression to promote the proliferation and tumorigenicity of endometrial cancer. J Cancer. (2020) 11:5612–22. doi: 10.7150/jca.46097 PMC7477457 32913456

[182] Zhang L Wan Y Zhang Z Jiang Y Lang J Cheng W. Fto demethylates M6a modifications in hoxb13 mrna and promotes endometrial cancer metastasis by activating the wnt signalling pathway. RNA Biol. (2021) 18:1265–78. doi: 10.1080/15476286.2020.1841458 PMC8354663 33103587

[183] Fan L Lin Y Lei H Shu G He L Yan Z. A newly defined risk signature, consisting of three M(6)a rna methylation regulators, predicts the. Aging (Albany NY). (2020) 12:18453–75. doi: 10.2139/ssrn.3514606 PMC7585096 32950970

[184] Zhu W Zhao L Kong B Liu Y Zou X Han T. The methylation modification of M6a regulators contributes to the prognosis of ovarian cancer. Ann Transl Med. (2022) 10:59. doi: 10.21037/atm-21-6462 35282121 PMC8848366

[185] Jiao J Jiang L Luo Y. N6-methyladenosine-related rna signature predicting the prognosis of ovarian cancer. Recent Pat Anticancer Drug Discov. (2021) 16:407–16. doi: 10.2174/1574892816666210615164645 34137363

[186] Wei Q Yang D Liu X Zhao H Yang Y Xu J. Exploration of the role of M(6) a rna methylation regulators in Malignant progression and clinical prognosis of ovarian cancer. Front Genet. (2021) 12:650554. doi: 10.3389/fgene.2021.650554 34149801 PMC8209520

[187] Liu Y Li L Li Y Zhao X. Research progress on tumor-associated macrophages and inflammation in cervical cancer. BioMed Res Int. (2020) 2020:6842963. doi: 10.1155/2020/6842963 32083131 PMC7011341

[188] Ferrall L Lin KY Roden RBS Hung CF Wu TC. Cervical cancer immunotherapy: facts and hopes. Clin Cancer Res. (2021) 27:4953–73. doi: 10.1158/1078-0432.CCR-20-2833 PMC8448896 33888488

[189] Tewari KS Monk BJ. New strategies in advanced cervical cancer: from angiogenesis blockade to immunotherapy. Clin Cancer Res. (2014) 20:5349–58. doi: 10.1158/1078-0432.Ccr-14-1099 25104084

[190] Pan J Xu L Pan H. Development and validation of an M6a rna methylation regulator-based signature for prognostic prediction in cervical squamous cell carcinoma. Front Oncol. (2020) 10:1444. doi: 10.3389/fonc.2020.01444 32974164 PMC7472601

[191] Wang Q Guo X Li L Gao Z Su X Ji M. N(6)-methyladenosine mettl3 promotes cervical cancer tumorigenesis and warburg effect through ythdf1/hk2 modification. Cell Death Dis. (2020) 11:911. doi: 10.1038/s41419-020-03071-y 33099572 PMC7585578

[192] Li Z Peng Y Li J Chen Z Chen F Tu J. N(6)-methyladenosine regulates glycolysis of cancer cells through pdk4. Nat Commun. (2020) 11:2578. doi: 10.1038/s41467-020-16306-5 32444598 PMC7244544

[193] Huang C Liang J Lin S Wang D Xie Q Lin Z. N(6)-methyladenosine associated silencing of mir-193b promotes cervical cancer aggressiveness by targeting ccnd1. Front Oncol. (2021) 11:666597. doi: 10.3389/fonc.2021.666597 34178650 PMC8222573

[194] Ni HH Zhang L Huang H Dai SQ Li J. Connecting mettl3 and intratumoural cd33(+) mdscs in predicting clinical outcome in cervical cancer. J Transl Med. (2020) 18:393. doi: 10.1186/s12967-020-02553-z 33059689 PMC7565373

[195] Ji F Lu Y Chen S Lin X Yu Y Zhu Y. M(6)a methyltransferase mettl3-mediated lncrna foxd2-as1 promotes the tumorigenesis of cervical cancer. Mol Ther Oncolytics. (2021) 22:574–81. doi: 10.1016/j.omto.2021.07.004 PMC8450180 34589576

[196] Zheng H Zheng WJ Wang ZG Tao YP Huang ZP Yang L. Decreased expression of programmed death ligand-L1 by seven in absentia homolog 2 in cholangiocarcinoma enhances T-cell-mediated antitumor activity. Front Immunol. (2022) 13:845193. doi: 10.3389/fimmu.2022.845193 35154166 PMC8828655

[197] Wang H Luo Q Kang J Wei Q Yang Y Yang D. Ythdf1 Aggravates the Progression of Cervical Cancer through M(6)a-Mediated up-Regulation of Ranbp2. Front Oncol. (2021) 11:650383. doi: 10.3389/fonc.2021.650383 33816306 PMC8017305

[198] Wangm T Ye B Zhang S Lei X Zhang D. Fto-stabilized lncrna hoxc13-as epigenetically upregulated. J BUON. (2021) 26:1279–91. 34564982

[199] Zhou S Bai ZL Xia D Zhao ZJ Zhao R Wang YY. Fto regulates the chemo-radiotherapy resistance of cervical squamous cell carcinoma (Cscc) by targeting beta-catenin through mrna demethylation. Mol Carcinog. (2018) 57:590–7. doi: 10.1002/mc.22782 29315835

[200] Ji F Lu Y Chen S Yu Y Lin X Zhu Y. Igf2bp2-modified circular rna circarhgap12 promotes cervical cancer progression by interacting M(6)a/foxm1 manner. Cell Death Discov. (2021) 7:215. doi: 10.1038/s41420-021-00595-w 34392306 PMC8364552

[201] Zhang Y Wang D Wu D Zhang D Sun M. Long noncoding rna kcnmb2-as1 stabilized by N(6)-methyladenosine modification promotes cervical cancer growth through acting as a competing endogenous rna. Cell Transplant. (2020) 29:963689720964382. doi: 10.1177/0963689720964382 33028109 PMC7784579

[202] Govindarajan M Wohlmuth C Waas M Bernardini MQ Kislinger T. High-throughput approaches for precision medicine in high-grade serous ovarian cancer. J Hematol Oncol. (2020) 13:134. doi: 10.1186/s13045-020-00971-6 33036656 PMC7547483

[203] Arora T MS Lekkala MR. Ovarian Cancer. Treasure Island (FL: StatPearls Publishing Copyright (2022).

[204] Nameki R Chang H Reddy J Corona RI Lawrenson K. Transcription factors in epithelial ovarian cancer: histotype-specific drivers and novel therapeutic targets. Pharmacol Ther. (2021) 220:107722. doi: 10.1016/j.pharmthera.2020.107722 33137377

[205] Baert T Ferrero A Sehouli J O'Donnell DM Gonzalez-Martin A Joly F. The systemic treatment of recurrent ovarian cancer revisited. Ann Oncol. (2021) 32:710–25. doi: 10.1016/j.annonc.2021.02.015 33675937

[206] Wang Z Meng F Zhong Z. Emerging targeted drug delivery strategies toward ovarian cancer. Advanced Drug Delivery Rev. (2021) 178:113969. doi: 10.1016/j.addr.2021.113969 34509574

[207] Hua W Zhao Y Jin X Yu D He J Xie D. Mettl3 promotes ovarian carcinoma growth and invasion through the regulation of axl translation and epithelial to mesenchymal transition. Gynecol Oncol. (2018) 151:356–65. doi: 10.1016/j.ygyno.2018.09.015 30249526

[208] Luo Y Sun X Xiong J. Characterization of M6a regulator-mediated methylation modification patterns and tumor microenvironment infiltration in ovarian cancer. Front Cell Dev Biol. (2021) 9:794801. doi: 10.3389/fcell.2021.794801 35087835 PMC8787330

[209] Yang Y Wei Q Tang Y Yuanyuan W Luo Q Zhao H. Loss of hnrnpa2b1 inhibits Malignant capability and promotes apoptosis via down-regulating lin28b expression in ovarian cancer. Cancer Lett. (2020) 475:43–52. doi: 10.1016/j.canlet.2020.01.029 32006618

[210] Xu F Li J Ni M Cheng J Zhao H Wang S. Fbw7 suppresses ovarian cancer development by targeting the N(6)-methyladenosine binding protein ythdf2. Mol Cancer. (2021) 20:45. doi: 10.1186/s12943-021-01340-8 33658012 PMC7927415

[211] Zhang Y Qiu J-G Jia X-Y Ke Y Zhang M-K Stieg D. Mettl3-mediated N6-methyladenosine modification and hdac5/yy1 promote iffo1 downregulation in tumor development and chemo-resistance. Cancer Lett. (2023) 553:215971. doi: 10.1016/j.canlet.2022.215971 36257380

[212] Yu HL Ma XD Tong JF Li JQ Guan XJ Yang JH. Wtap is a prognostic marker of high-grade serous ovarian cancer and regulates the progression of ovarian cancer cells. Onco Targets Ther. (2019) 12:6191–201. doi: 10.2147/OTT.S205730 PMC6689666 31496724

[213] Wang Y Chen Z. Long noncoding rna uba6-as1 inhibits the Malignancy of ovarian cancer cells via suppressing the decay of uba6 mrna. Bioengineered. (2022) 13:178–89. doi: 10.1080/21655979.2021.2011640 PMC8805991 34951345

[214] Zhao L Kong X Zhong W Wang Y Li P. Fto accelerates ovarian cancer cell growth by promoting proliferation, inhibiting apoptosis, and activating autophagy. Pathol Res Pract. (2020) 216:153042. doi: 10.1016/j.prp.2020.153042 32825930

[215] Huang H Wang Y Kandpal M Zhao G Cardenas H Ji Y. (6)-methyladenosine modifications inhibit ovarian cancer stem cell self-renewal by blocking camp signaling. Cancer Res. (2020) 80:3200–14. doi: 10.1158/0008-5472.CAN-19-4044 PMC7442742 32606006

[216] Jiang X Liu B Nie Z Duan L Xiong Q Jin Z. The role of M6a modification in the biological functions and diseases. Signal Transduction Targeted Ther. (2021) 6:74. doi: 10.1038/s41392-020-00450-x PMC7897327 33611339

[217] Jiang Y Wan Y Gong M Zhou S Qiu J Cheng W. Rna demethylase alkbh5 promotes ovarian carcinogenesis in a simulated tumour microenvironment through stimulating nf-kappab pathway. J Cell Mol Med. (2020) 24:6137–48. doi: 10.1111/jcmm.15228 PMC7294121 32329191

[218] Zhu H Gan X Jiang X Diao S Wu H Hu J. Alkbh5 inhibited autophagy of epithelial ovarian cancer through mir-7 and bcl-2. J Exp Clin Cancer Res. (2019) 38:163. doi: 10.1186/s13046-019-1159-2 30987661 PMC6463658

[219] Sun R Yuan L Jiang Y Wan Y Ma X Yang J. Alkbh5 activates fak signaling through M6a demethylation in itgb1 mrna and enhances tumor-associated lymphangiogenesis and lymph node metastasis in ovarian cance. Theranostics. (2023) 13:833–48. doi: 10.7150/thno.77441 PMC9830429 36632222

[220] Muller S Glass M Singh AK Haase J Bley N Fuchs T. Igf2bp1 promotes srf-dependent transcription in cancer in a M6a- and mirna-dependent manner. Nucleic Acids Res. (2019) 47:375–90. doi: 10.1093/nar/gky1012 PMC6326824 30371874

[221] Bley N Schott A Muller S Misiak D Lederer M Fuchs T. Igf2bp1 is a targetable src/mapk-dependent driver of invasive growth in ovarian cancer. RNA Biol. (2021) 18:391–403. doi: 10.1080/15476286.2020.1812894 32876513 PMC7951963

[222] Wang S Li Z Zhu G Hong L Hu C Wang K. Rna-binding protein igf2bp2 enhances circ_0000745 abundancy and promotes aggressiveness and stemness of ovarian cancer cells via the microrna-3187-3p/erbb4/pi3k/akt axis. J Ovarian Res. (2021) 14:154. doi: 10.1186/s13048-021-00917-7 34774079 PMC8590297

